# Strand-selective base editing of human mitochondrial DNA using mitoBEs

**DOI:** 10.1038/s41587-023-01791-y

**Published:** 2023-05-22

**Authors:** Zongyi Yi, Xiaoxue Zhang, Wei Tang, Ying Yu, Xiaoxu Wei, Xue Zhang, Wensheng Wei

**Affiliations:** 1 Biomedical Pioneering Innovation Center, Peking-Tsinghua Center for Life Sciences, Peking University Genome Editing Research Center, State Key Laboratory of Protein and Plant Gene Research, School of Life Sciences, Peking University, Beijing, P.R. China; 2 Changping Laboratory, Beijing, P.R. China; 3 Academy for Advanced Interdisciplinary Studies, Peking University, Beijing, P.R. China

**Keywords:** Genetic engineering, Targeted gene repair

## Abstract

A number of mitochondrial diseases in humans are caused by point mutations that could be corrected by base editors, but delivery of CRISPR guide RNAs into the mitochondria is difficult. In this study, we present mitochondrial DNA base editors (mitoBEs), which combine a transcription activator-like effector (TALE)-fused nickase and a deaminase for precise base editing in mitochondrial DNA. Combining mitochondria-localized, programmable TALE binding proteins with the nickase MutH or Nt.BspD6I(C) and either the single-stranded DNA-specific adenine deaminase TadA8e or the cytosine deaminase APOBEC1 and UGI, we achieve A-to-G or C-to-T base editing with up to 77% efficiency and high specificity. We find that mitoBEs are DNA strand-selective mitochondrial base editors, with editing results more likely to be retained on the nonnicked DNA strand. Furthermore, we correct pathogenic mitochondrial DNA mutations in patient-derived cells by delivering mitoBEs encoded in circular RNAs. mitoBEs offer a precise, efficient DNA editing tool with broad applicability for therapy in mitochondrial genetic diseases.

## Abstract (web summary)

Mitochondrial point mutations are corrected in human cells using a transcription activator-like effector (TALE)-based system.

## Main

Mitochondrial DNA (mtDNA) mutations are associated with many human diseases, and around 95% of these are point mutations that could potentially be corrected using base editing approaches. Therefore, there is a high demand for technologies that enable mtDNA base editing, which could aid in understanding the underlying mechanisms of pathogenesis and developing cures for these diseases. Although the CRISPR system has been widely used for nuclear genome base editing^1,2^, it is currently impractical to apply this system for editing the mitochondrial genome due to the absence of an effective method for delivering guide RNA into this organelle^3^.

Most human cells with mitochondrial disease have heteroplasmic mtDNA that exists in multiple copies. Mutant mtDNA coexists with wild-type mtDNA, and the ratio of wild-type to mutant mtDNA often correlates with the severity of the clinical phenotype^4^. Researchers have fused mitochondrial targeting sequences with RNA-free programmable nucleases, such as zinc-finger nucleases and transcription activator-like effector (TALE) nucleases (TALENs), to achieve targeted degradation of mutant mtDNA and increase the proportion of wild-type mtDNA^5–8^. However, these approaches are not suitable for treating mitochondrial diseases involving homogeneous mutations and do not support the introduction of new sequence changes. Recently, DdCBEs (DddA-derived cytosine base editors)^9–11^ and TALEDs (transcription-activator-like effector-linked deaminases)^12^ have been developed to achieve C-to-T and A-to-G conversions, respectively, in mtDNA. DdCBEs involve the fusion of split DddA halves, TALE array proteins and uracil glycosylase inhibitor (UGI)^9–11^, whereas TALEDs combine TALE, DddA and deaminase to achieve A-to-G editing in mitochondria^12^.

Both DdCBEs and TALEDs perform base deamination on both strands of double-stranded DNA (dsDNA) within the editing window. However, single TALE binding can lead to off-target effects in the split DddA halves. In addition, DddA’s direct or indirect interaction with CTCF can result in a broad range of off-target effects on the nuclear genome^13^.

Given that deamination of either C or A should not occur while in the state of base pairing^1^, we hypothesized that introducing single-stranded DNA (ssDNA) around the target loci could enable targeted deamination of mtDNA. To this end, we developed a method for efficient and accurate mtDNA base editing by combining a deaminase and nickase.

## Results

### Insufficiency of TALE–TadA8e-V106W alone to facilitate the editing of mtDNA

**Fig. 1:**
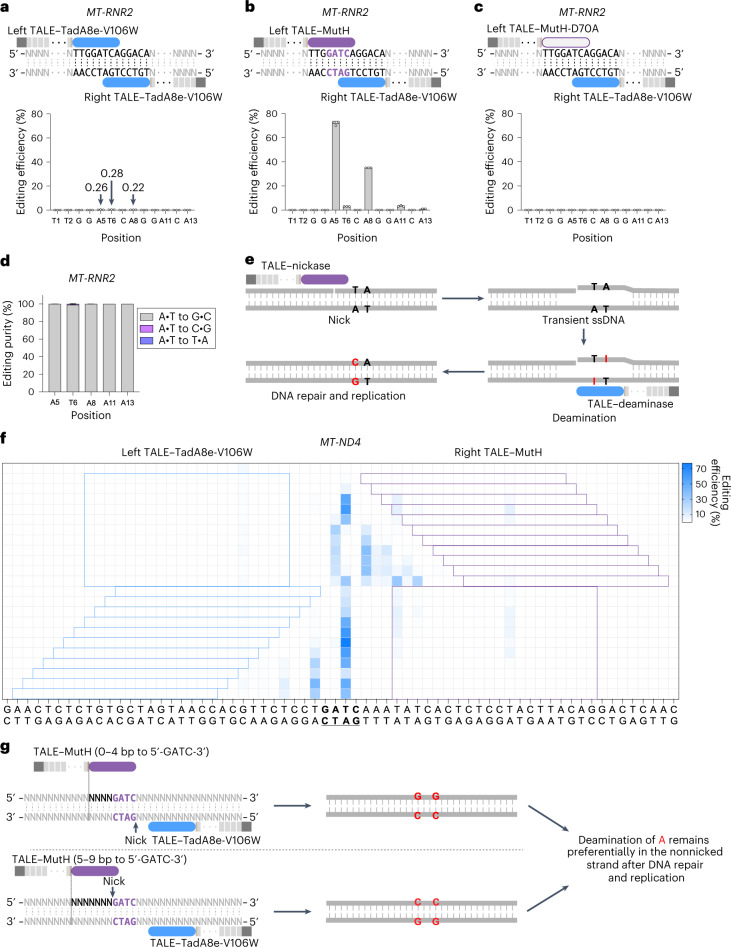
Strand-biased editing of mtDNA using programmable nickase and deaminase. **a**, Mitochondrial A-to-G editing efficiency of HEK293T cells treated with paired TALE–TadA8e-V106W at *the MT-RNR2* site. **b**, Mitochondrial A-to-G editing efficiency of HEK293T cells treated with left TALE–MutH and right TALE–TadA8e-V106W at the *MT-RNR2* site. **c**, Mitochondrial A-to-G editing efficiency of HEK293T cells treated with left TALE–MutH-D70A and right TALE–TadA8e-V106W at the *MT-RNR2* site. **d**, Product distributions at the *MT-RNR2* site in (**b**). **e**, A speculative model for improving the editing efficiency of mtDNA by combining nickase with deaminase (take TadA8e-V106W as an example in this model). TALE–nickase binds the target DNA and nicks the dsDNA. The nicked dsDNA may be prone to form ssDNA structures. TALE–TadA8e-V106W binds the target DNA and efficiently deaminates the adenine(s) on the resulting ssDNA. The resulting inosine(s) can be converted permanently to guanine(s) after DNA repair or DNA replication. **f**, Editing efficiency of mitoABE^MutH^ with MutH and TadA8e-V106W at different distances from 5′-GATC-3′ at *MT-ND4*. **g**, A speculative model for mitoABE^MutH^. When the distance between TALE–MutH and 5′-GATC-3′ is 0–4 bp, the opposite strand is nicked, thus causing editing of the TALE–MutH recognition strand; when the distance between TALE–MutH and 5′-GATC-3′ is 5–9 bp, the TALE–MutH recognition strand is nicked, thus causing editing of the opposite strand. For **a**–**d**, data are presented as mean ± s.d. of *n* = 3 independent biological replicates; for **f**, the mean values from *n* = 3 independent biological replicates are shown. The blue rectangular blocks represent the left TALE array binding sequences, and the purple rectangular blocks represent the right TALE array binding sequences. In **a**–**c**, **e** and **g**, the blue rounded rectangle represents TadA8e-V106W, the purple rounded rectangle represents nickase and the gray rectangle represents TALE.

**Extended Data Fig. 1:**
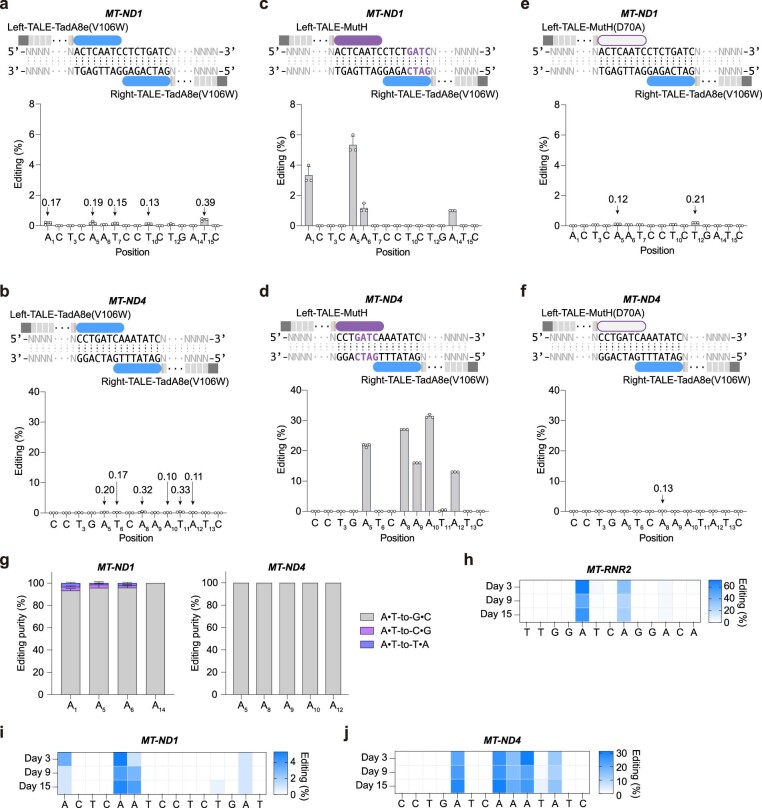
Improving the mtDNA editing efficiency of TadA8e(V106W) by introducing nickase. **a** and **b**, Mitochondrial A-to-G editing efficiency of HEK293T cells treated with paired TALE-TadA8e(V106W) at *MT-ND1* (**a**) and *MT-ND4* (**b**). **c** and **d**, Mitochondrial A-to-G editing efficiency of HEK293T cells treated with Left-TALE-MutH and Right-TALE-TadA8e(V106W) at *MT-ND1* (**c**) and *MT-ND4* (**d**). **e** and **f**, Mitochondrial A-to-G editing efficiency of HEK293T cells treated with Left-TALE-MutH(D70A) and Right-TALE-TadA8e(V106W) at *MT-ND1* (**e**) and *MT-ND4* (**f**). **g**, Product distributions at the two sites in (**c**) and (**d**). In **a**-**f**, the blue rounded rectangle represents TadA8e(V106W), the purple rounded rectangle represents MutH, and the gray rectangle represents TALE. **h-j**, Time-course analysis of editing efficiencies at *MT-RNR2* (**h**), *MT-ND1* (**i**) and *MT-ND4* (**j**). For **a**-**g**, data are presented as mean values ± s.d. of n = 3 independent biological replicates. For **h-j**, all data points from n = 3 biologically independent replicates are shown.

TadA8e-V106W is an engineered deoxyadenosine deaminase that is commonly fused with Cas protein to perform adenosine-to-inosine (recognized as guanine) editing on nuclear DNA^14^. To investigate the potential for mitochondrial A-to-G editing, we fused the TadA8e-V106W protein with the appropriate TALE array and mitochondrial targeting sequence. We detected a very low level of editing at all three targeting sites, *MT-ND1*, *MT-ND4* and *MT-RNR2*, with an editing rate of up to 0.39%, which is barely above the deep sequencing error (>0.10%) (Fig. 1a and Extended Data Fig. 1a,b). Because TadA is an essential tRNA-specific adenosine deaminase originating from *Escherichia coli*^15^, it is unsurprising that TadA8e-V106W enables mtDNA editing. Nevertheless, TadA8e-V106W alone was unable to induce efficient deamination because TALE cannot unravel the DNA double helix^16,17^, and this deaminase preferentially acts on ssDNA^1,18^, even after optimization through multiple rounds of mutations^14^. For base editing, the Cas9 and single-guide RNA complex unravels the DNA double helix at the target site to expose an ssDNA structure that serves as the substrate for ssDNA deaminase (for example, TadA8e-V106W or rAPOBEC1)^1^. We hypothesized that generating an ssDNA structure at the target loci might unleash the full power of TadA8e-V106W’s deaminase activity.

### Efficient targeted A-to-G editing of mtDNA by a combination of TALE–TadA8e-V106W and TALE–nickase

To investigate whether deamination can occur on ssDNA, we used nickase to cleave only one strand. Our initial attempt involved using MutH, a sequence-specific (5′-GATC-3′) nickase encoded by *E. coli* that is involved in initiating mismatch repair to remove nucleotides misincorporated by DNA polymerase^19^. Wild-type MutH can nick unmethylated DNA strands in unmethylated or hemimethylated DNA^20^. In previous work, Gabsalilow et al.^21^ achieved strand-specific nicking of DNA by fusing TALE and MutH, and the strand-specific nick outcome depended on the distance between the TALE-array binding sequence and MutH recognition site. By fusing MutH with an appropriate TALE array, we introduced TALE–MutH and TALE–TadA8e-V106W in pairs to target the loci *MT-ND1*, *MT-ND4* and *MT-RNR2*. We observed high levels of targeted A-to-G editing at all three loci, with a maximum efficiency of up to 77% (Fig. 1b and Extended Data Fig. 1c,d). Nickase activity was crucial for editing activity because the endonuclease-inactivated MutH mutant^22^, TALE–MutH-D70A, failed to enable any base editing when paired with its corresponding TALE–TadA8e-V106W (Fig. 1c and Extended Data Fig. 1e,f). These results indicated that TALE–nickase may generate ssDNA that exposed adenines, which serve as more suitable substrates for TadA8e-V106W. We thus created a new, to our knowledge, mitochondrial A-to-G base editing system, designated mitoABE^MutH^. As expected, the dominant editing outcome was A•T to G•C, with a product purity of over 95% at the *MT-ND1* locus (Extended Data Fig. 1g) and close to 100% at both the *MT-RNR2* and *MT-ND4* loci (Fig. 1d and Extended Data Fig. 1g). We confirmed the durability of mtDNA editing results in HEK293T cells over 15 days at three target loci (Extended Data Fig. 1h–j).

On the basis of the above results, we proposed a theoretical model to explain how the TALE–nickase and TALE–deaminase combination enables the base editing of mtDNA. By nicking the target site, TALE–nickase generates transient ssDNA and consequently allows a TALE–deaminase (such as TALE–TadA8e-V106W) to deaminate adenine(s) on the ssDNA. After repair and replication of mtDNA, the deamination of adenine(s) probably remains in one strand, leading to strand-biased A-to-G conversion (Fig. 1e).

### Strand-biased editing of mitoABEs

**Extended Data Fig. 2:**
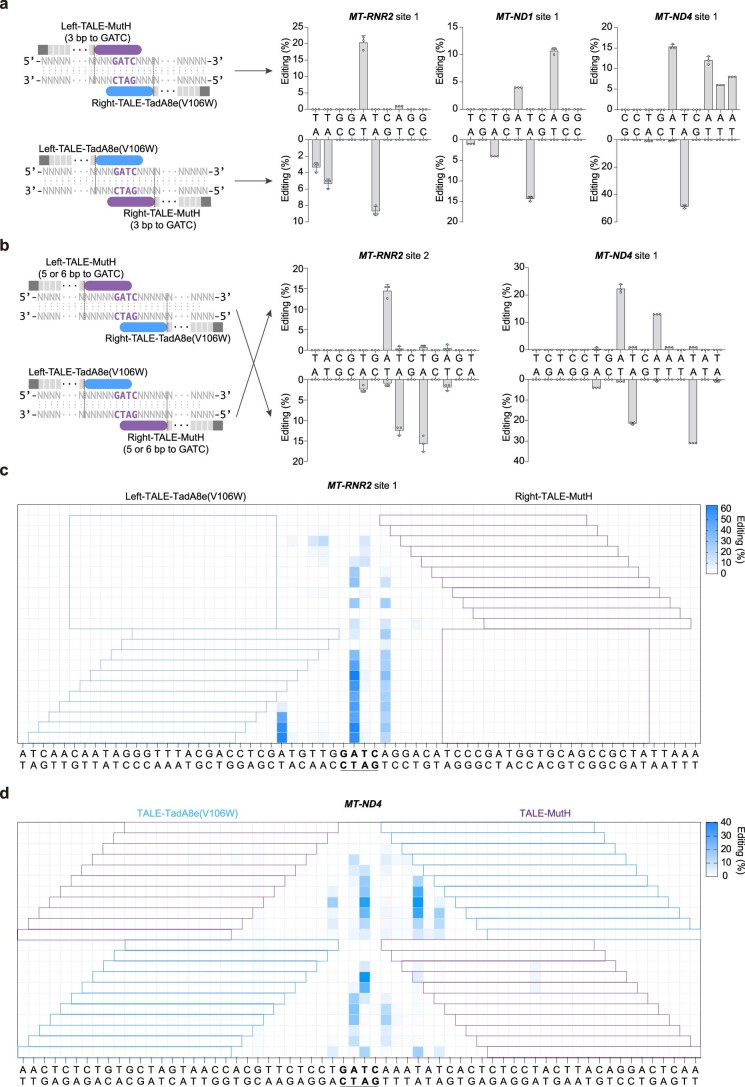
Strand-biased editing of mitochondrial DNA using mitoABE^MutH^. **a**, Different orientations of mitoABE^MutH^ when the 3 bp distance from 5’-GATC-3’ and the adenines on different strands are edited at *MT-RNR2* site 1, *MT-ND1* site 1 and *MT-ND4* site 1. Top, Left-TALE-MutH and Right-TALE- TadA8e(V106W). Bottom, Left-TALE-TadA8e(V106W) and Right-TALE- MutH. **b**, Different orientations of mitoABE^MutH^ when the 5 or 6 bp distance from 5’-GATC-3’ and the adenines on different strands are edited at *MT-RNR2* site 2 and *MT-ND4* site 1. Top, Left-TALE-TadA8e(V106W) and Right-TALE-MutH. Bottom, Left-TALE-MutH and Right-TALE-TadA8e(V106W). For **a** and **b**, data are presented as mean values ± s.d. of n = 3 independent biological replicates. In **a** and **b**, the blue rounded rectangle represents TadA8e(V106W), the purple rounded rectangle represents nickase, and the gray rectangle represents TALE. **c** and **d**, Editing efficiency of mitoABE^MutH^ with MutH and TadA8e(V106W) at different distances from 5’-GATC-3’ at *MT-RNR2* site 1 (**c**) and *MT-ND4* (**d**). For **c** and **d**, the mean values from n = 3 biologically independent replicates are shown.

mitoABE^MutH^-enabled adenine editing occurred preferentially on the top strands of the three target loci (*MT-ND1*, *MT-ND4* and *MT-RNR2*) (Fig. 1b and Extended Data Fig. 1c,d). Despite the presence of multiple adjacent Ts next to the edited A within the editing windows, the A on its opposite strand appeared unedited (Extended Data Fig. 1c,d) or only barely edited (Fig. 1b). These results suggest that the editing of mitoABE^MutH^ may be strand specific. To verify this notion, we switched the TALE arrays for each pair of TALE–MutH and TALE–TadA8e-V106W and found that the strand-biased edits were reciprocally switched (Extended Data Fig. 2a,b). In *MT-RNR2* site 1, *MT-ND1* site 1 and *MT-ND4* site 1, we placed the MutH nicking sequence (5′-GATC-3′) in the center of the editing window, 3 base pairs (bp) away from each TALE (Extended Data Fig. 2a). In this case, editing occurred mainly on the top DNA strand using left TALE–MutH and right TALE–TadA8e-V106W, whereas editing occurred mainly on the bottom strand when switching the positions of TALE–MutH and TALE–TadA8e-V106W (Extended Data Fig. 2a). In *MT-RNR2* site 2 and *MT-ND4* site 1, the MutH nicking sequence is 5 or 6 bp away from each end of the editing window (Extended Data Fig. 2b), and editing occurs mainly on the bottom DNA strand with left TALE–MutH and right TALE–TadA8e-V106W. The top DNA strand became the A-edited strand after switching the MutH and TadA8e-V106W TALEs (Extended Data Fig. 2b). On the basis of these results, we speculated that strand-biased editing is related to the strand selection of nicking, which is, in turn, related to the number of bases between TALE binding and the MutH recognition motif (5′-GATC-3′).

Next, we fixed the position of left TALE–TadA8e-V106W and shifted the position of right TALE–MutH from 0 to 10 bp away from the nicking sequence (5′-GATC-3′) successively by 1 bp (Fig. 1f). Editing occurred mainly on the bottom DNA strand at both *MT-ND4* and *MT-RNR2* site 1 when the distance between TALE binding and the MutH nick motif was within 0 to 4 bp, and editing occurred mainly on the top DNA strand when the distance between TALE binding and the MutH nick motif was between 5 and 9 bp (Fig. 1f and Extended Data Fig. 2c). In contrast, when we fixed TALE–MutH and changed the position of TALE–TadA8e-V106W, the A-edited strand did not change, the editing windows only widened gradually (Fig. 1f).

We conducted experiments to determine whether the orientation of MutH relative to TALE affected the strand-biased editing of mitoABE^MutH^. Because the MutH nick motif (5′-GATC-3′) is a palindromic sequence, we suspected the fusion of MutH with left TALE or right TALE would have the same effect. By gradually widening the editing window at the *MT-ND4* site (Extended Data Fig. 2d), the orientation-switched TALE–MutH still showed the distance-dependent, edited-strand preference in editing outcome. Editing was successful between the 8- and 24-bp editing windows at this site (Extended Data Fig. 2d). Therefore, we presumed a working model for mitoABE^MutH^ in which TALE–MutH nicks the strand opposite its binding strand when the distance between TALE binding and MutH nick motif is 0–4 bp, and it nicks the same strand when the distance is 5–9 bp, which is consistent with previous work^21^. Nicking generates ssDNAs, and all adenines on ssDNA are subjected to TadA8e-V106W-mediated deamination within the window. After repair and DNA replication, most deaminated A on the nonnicked strand was retained (Fig. 1g). These findings indicated that TadA8e-V106W editing outcome depends on which strand is nicked by TALE–nickase, and adenine deamination is predominantly preserved on the nonnicked strand. We also observed that the linker sequences between TALE and MutH had no effect on the strand-biased editing of mitoABE^MutH^ in our experiments (Supplementary Fig. 1a–d).

### Expanded targeting scope of mitoABE^MutH^ by site-directed mutations

**Fig. 2:**
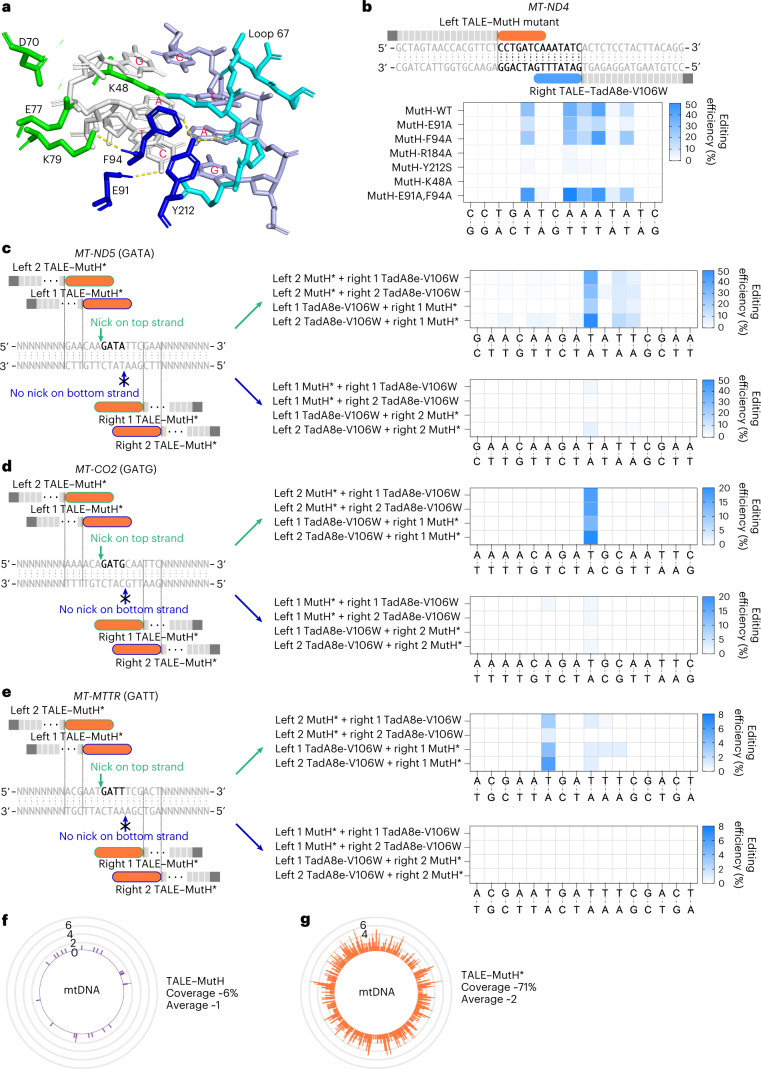
Broadened editing to 5′-GAT-3′ by introducing mutations to MutH. **a**, Crystal structure of key amino acids of MutH interacting with unmethylated 5′-GATC-3′ (PDB: 2AOQ). **b**, Editing efficiency of MutH mutants (including K48A, E91A, F94A, R184A, Y212S and double mutation of E91A and F94A) combined with TadA8e-V106W at the 5′-GATC-3′ position. **c**–**e**, The editing efficiencies of target regions at the 5′-GATA-3′ (**c**), 5′-GATG-3′ (**d**) and 5′-GATT-3′ positions (**e**) with different mitoABE^MutH*^ orientations and distances. In **b**–**e**, the mean values from *n* = 3 biologically independent replicates are shown. **f**,**g**, The designable targeting range of TALE–MutH (**f**) and TALE–MutH* (**g**) in human mitochondria. The numbers 0, 2, 4 and 6 indicate the frequency of MutH and MutH* recognition sequences within a 40-bp region. In **b**, **c**, **d** and **e**, the blue rounded rectangle represents TadA8e-V106W, the orange rounded rectangle represents the MutH mutant and the gray rectangle represents TALE. The borders of rounded rectangles of different colors (blue and green) represent TALE–MutH* designed to nick different strands. WT, wild type.

**Extended Data Fig. 3:**
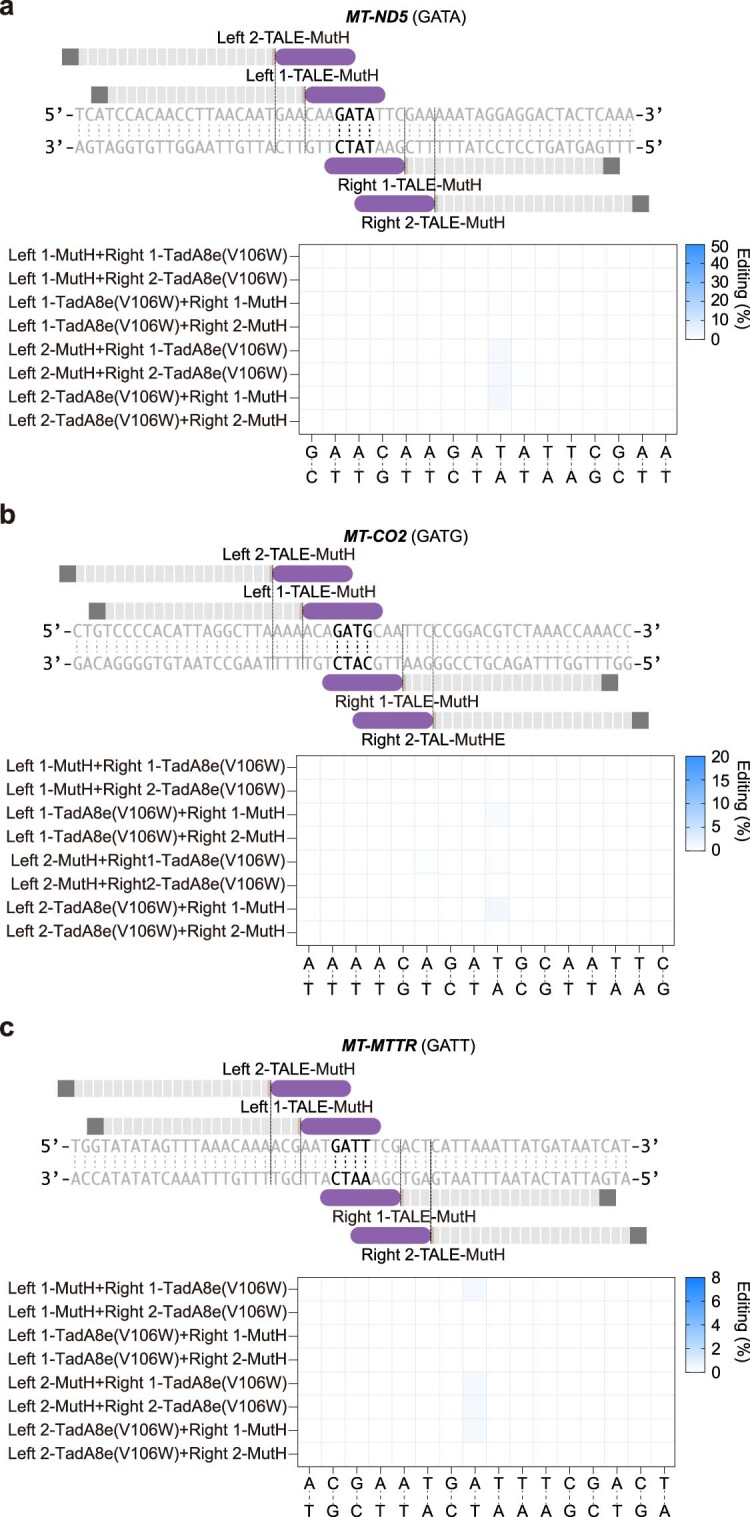
mitoABE^MutH^ un-enable efficient editing on non-GATC sites. The editing efficiencies of target regions at the 5’-GATA-3’ (**a**), 5’-GATG-3’ (**b**) and 5’-GATT-3’ (**c**) positions with different mitoABE^MutH^ orientations and distances to 5’-GATD-3’. **a**-**c**, the mean values from n = 3 biologically independent replicates are shown. In **a**-**c**, the purple rounded rectangle represents MutH, and the gray rectangle represents TALE.

The combination of TALE–MutH and TALE–TadA8e-V106W enables targeted strand-biased editing of mtDNA. However, MutH requires a specific sequence for nicking (5′-↓GATC-3′), limiting the editing scope of mitoABE^MutH^. Based on structural information (PDB: 2AOQ)^22^, we attempted to expand the editing scope of mitoABE^MutH^ by introducing point mutation(s) to MutH. We found that K48A, R184A and Y212S abolished the editing activity of mitoABE^MutH^ at the *MT-ND4* site (Fig. 2a,b). In MutH, F94 helps loop 67 (amino acid residues 184–190) to make sequence-specific interactions with 5′-GATC-3′, and F91 interacts with the cytosine in 5′-GATC-3′ (ref. ^22^) (Fig. 2a). The E91A or F94A variant maintained the editing activity of mitoABE^MutH^, and the combination of these two mutations enhanced editing efficiency at the *MT-ND4* site (Fig. 2b). We designated this special type of MutH mutant harboring E91A and F94A MutH*. We then investigated whether MutH* could generate nicks at 5′-GATD-3′ (D stands for A, T or G) sites and become a new type of mtDNA editing tool, mitoABE^MutH*^. By targeting three loci, *MT-ND5*, *MT-CO2* and *MT-MTTR*, which, respectively contain 5′-GATA-3′, 5′-GATG-3′ and 5′-GATT-3′ sequences on the top strand, we confirmed that mitoABE^MutH*^ indeed worked as an effective editing tool that generated bottom-strand edits at all three sites (Fig. 2c–e). Importantly, none of these sites could be edited by mitoABE^MutH^ because of the absence of the MutH motif (Extended Data Fig. 3a–c).

The MutH motif, 5′-GATC-3′, is a palindromic sequence; thus, TALE–MutH* (5′-GATD-3′) can only nick the top strand by the 5′-guanine in certain designs, and only adenine edits on the bottom strand are retained (Fig. 2c–e). On the other hand, TALE–MutH cannot nick 5′-GATD-3′, resulting in no edits (Extended Data Fig. 3a–c). In addition, MutH* yielded the best editing efficiency when placed 3 bp from 5′-GATD-3′ at the right end, probably due to its high nicking efficiency (Fig. 2c–e). These results confirmed the presumed working principle of mitoABE^MutH^, and TALE–MutH* indeed expands its editing scope. There are 23 recognition sites (5′-GATC-3′) of MutH in human mtDNA, but MutH* (5′-GATN-3′) has 485 sites. We speculate that TALE–TadA8e-V106W could function within a range of 20-bp upstream and downstream of the nick position. Based on this, we estimated the proportion of designable mitoABEs in the human mitochondrial genome. TALE–MutH has a designable targeting range of only ~6% of the mitochondrial genome, whereas TALE–MutH* has a range of ~71%, with an average of two 5′-GATN-3′ sites per 40 bp in the mitochondrial genome (Fig. 2f,g). Despite expanding the editing scope of mitoABEs greatly, there are still many sequences that cannot be edited by mitoABEs. Therefore, further expansion of the editing scope is necessary.

### Screening for alternative nickases with no sequence-context constraints

**Extended Data Fig. 4:**
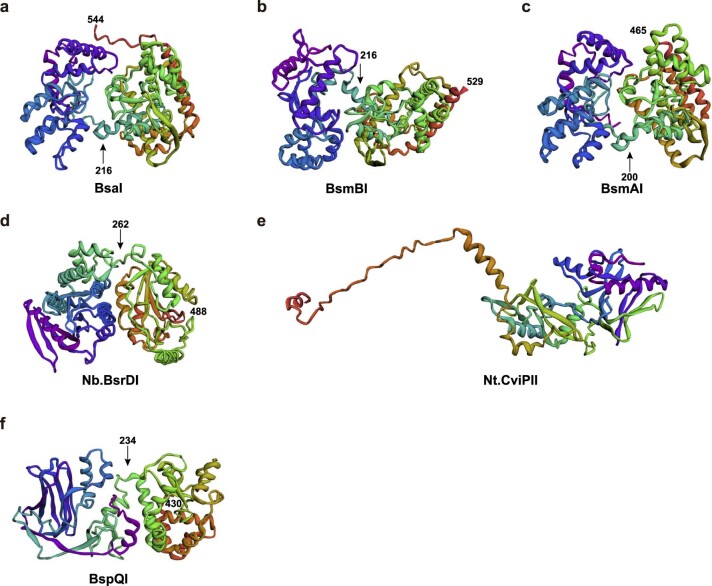
The predicted structure of the screened nickases. **a**, BsaI, **b**, BsmBI, **c**, BsmAI, **d**, Nb. BsrDI, **e**, Nt. CviPII, **f**, BspQI. The arrow indicates the segmentation location, and the C-terminus of the protein is the cleavage domain of the corresponding protein we selected. Full-length Nt.CviPII was used.

To further broaden the editable scope of mitoABEs, we tested multiple enzymes with potential nickase activity. As some nucleases have separate active centers for cutting dsDNA, mutation(s) inactivating one active center might convert the nuclease to nickase. In particular, the cleavage and recognition domains of type IIS restriction endonuclease are separable^23^, which makes it an ideal candidate of nickase for the half-deactivation cleavage domains. For enzymes without crystal structures, we attempted to predict their cleavage domains for engineering purposes^24^ (Extended Data Fig. 4). We began by replacing the MutH component of mitoABE^MutH^ with the naturally existing nickase Nt.BspD6I(C)^25^ and engineered nickases, such as FokI-FokI-D450A^26^, Nb.BsaI(C)-N441D/R442G^27^, Nt.BsaI(C)-R236D^27^, Nb.BsmBI(C)-R438D^27^, Nt.BsmAI(C)-R221D^27^, Nb.BsrDI(C)^28^, Nt.CviPII (5′-↓CCD-3′)^29^, BspQI(C)^30^, N.AlwI(C)^31^ and I-TevI (5′-CNNN↓G-3′)^32^ to verify whether any of these enzymes nicks DNA when fused with an appropriate TALE array. We removed the recognition domains of all the enzymes mentioned above, except for Nt.CviPII (5′-↓CCD-3′) and I-TevI (5′-CNNN↓G-3′) because these two nickases recognize more extensive sequences. Our objective was to identify nickases that solely rely on the TALE array for recognition and do not possess recognition motifs. Therefore, we included only those candidates that do not have recognition sequences or have extensive recognition sequences in our system.

**Fig. 3:**
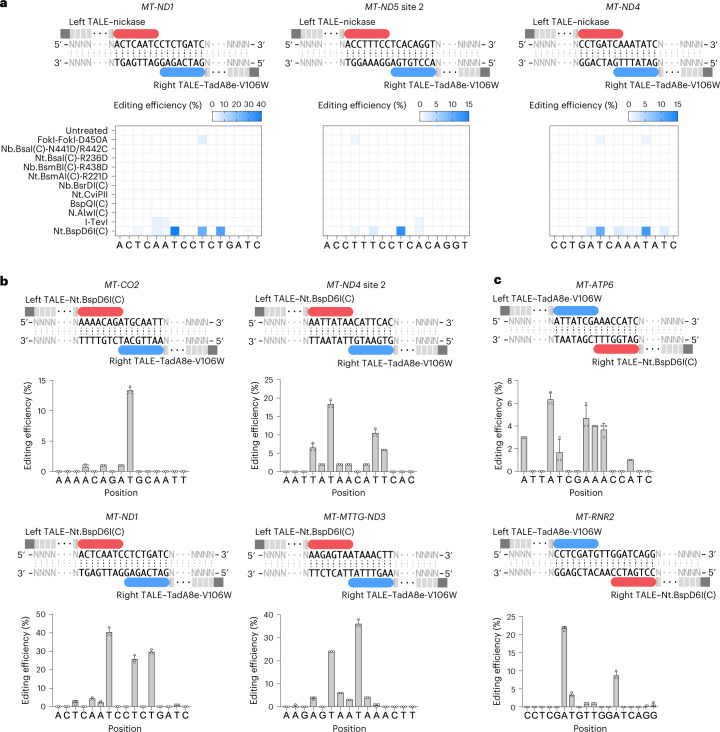
Screening nickases without recognition sequence restriction for mitoBEs. **a**, Nickases without sequence restriction or with extensive recognition sequences were screened for mitochondrial base editing at three sites. The mean values from *n* = 3 biologically independent replicates are shown. **b**, The editing efficiencies of different mitochondrial sites when left TALE–Nt.BspD6I(C) was combined with right TALE–TadA8e-V106W. **c**, The editing efficiencies of different mitochondrial sites when right TALE–Nt.BspD6I(C) combined with left TALE–TadA8e-V106W. For **b** and **c**, data are presented as mean values ±s.d. of *n* = 3 independent biological replicates. In **a**–**c**, the blue rounded rectangle represents TadA8e-V106W, the red rounded rectangle represents Nt.BspD6I(C) and the gray rectangle represents TALE.

By fusing the above nickases with left TALE, we tested their potential editing activities when teamed up with right TALE–TadA8e-V106W (Fig. 3a). The three editing sites *MT-ND1*, *MT-ND5* site 2 and *MT-ND4* were selected for testing. Among all TALE array-fused candidate nickases, the TALE–FokI-FokI-D450A, TALE–I-TevI and TALE–Nt.BspD6I(C) enabled editing on targeted sites when combined with TALE–TadA8e-V106W. TALE–Nt.BspD6I(C) yielded higher base editing activity at all three targeted sites (Fig. 3a). Nt.BspD6I is a nickase that can form a heterodimer with BspD6I (the small subunit, 20 kDa) and function as a restriction endonuclease called R.BspD6I^25^. Nt.BspD6I(C), which we used to fuse with TALE, is only the C-terminal cleavage domain (382–604 amino acids). In comparison with TALE–MutH, TALE–Nt.BspD6I(C) showed lower strand preference at the *MT-ND4* site (Fig. 3a and Extended Data Fig. 1d), possibly due to its imprecise nick on dsDNA. We named this editing tool mitoABE^Nt.BspD6I(C)^.

To further characterize the editing pattern of mitoABE^Nt.BspD6I(C)^, we applied mitoABE^Nt.BspD6I(C)^ to target more diverse mtDNA sequences. mitoABE^Nt.BspD6I(C)^ reached up to ~40% editing efficiency at some of these sites (Fig. 3b). In addition, when the TALEs of TALE–Nt.BspD6I(C) and TALE–TadA8e-V106W were switched, the edited strand was switched reciprocally (Fig. 3b,c). The linker sequences between TALE and Nt.BspD6I(C) did not affect the editing features of mitoABE^Nt.BspD6I(C)^ (Supplementary Fig. 1e–h). From all tested sites, we speculated that TALE–Nt.BspD6I(C) produced the nick on the same DNA strand recognized by itself, resulting in the editing of adenine(s) in the strand recognized by TALE–TadA8e-V106W.

### Mitochondrial C-to-T editing via cytosine deaminase

**Fig. 4:**
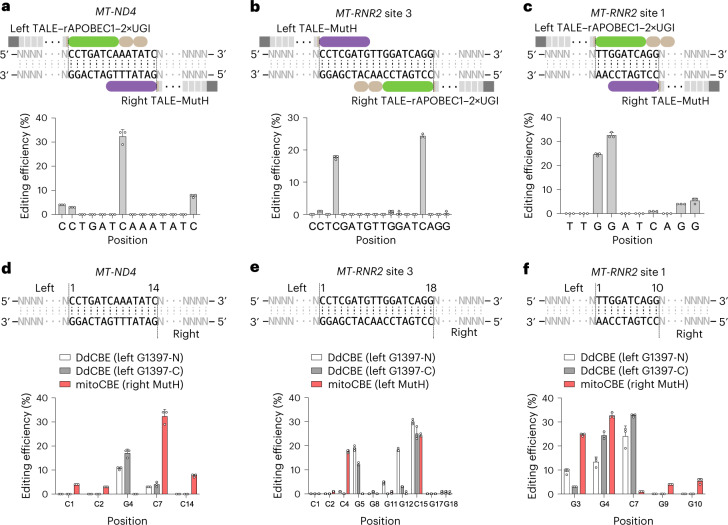
Mitochondrial C-to-T base editing by nickase combined with rAPOBEC1 and UGI. **a**–**c**, The editing efficiency of mitoCBEs at *MT-ND4* (**a**), *MT-RNR2* site 3 (**b**) and *MT-RNR2* site 1 (**c**). The green rounded rectangle represents rAPOBEC1, the brown rounded rectangle represents UGI, the purple rounded rectangle represents MutH and the gray rectangle represents TALE. **d**–**f**, Comparison of the editing profiles of mitoCBEs and DdCBEs at *MT-ND4* (**d**), *MT-RNR2* site 3 (**e**) and *MT-RNR2* site 1 (**f**). For **a**–**f**, data are presented as mean values ±s.d. of *n* = 3 independent biological replicates.

With the success of mitoABEs, we speculated that such a strategy could be extended to other types of deaminases, including rAPOBEC1, which converts C to T on ssDNA^1^. By replacing TadA8e-V106W with rAPOBEC1-fused UGI^33^, we were able to achieve mitochondrial C-to-T editing using the combination of TALE–rAPOBEC1–2×UGI and TALE–MutH, with a maximum editing efficiency of ~30% (Fig. 4a–c). Similar to mitoABE^MutH^, mitochondrial C-to-T editing, designated mitoCBE^MutH^, also displayed strand preference, with the top strands edited for *MT-ND4* and *MT-RNR2* site 3 (Fig. 4a,b), and the bottom strand edited for *MT-RNR2* site 1 (Fig. 4c). In contrast, editing of DdCBEs was not biased toward a specific strand at these three sites (Fig. 4d–f).

### Monomeric mitoBEs for base editing on mitochondrial genome

**Extended Data Fig. 5:**
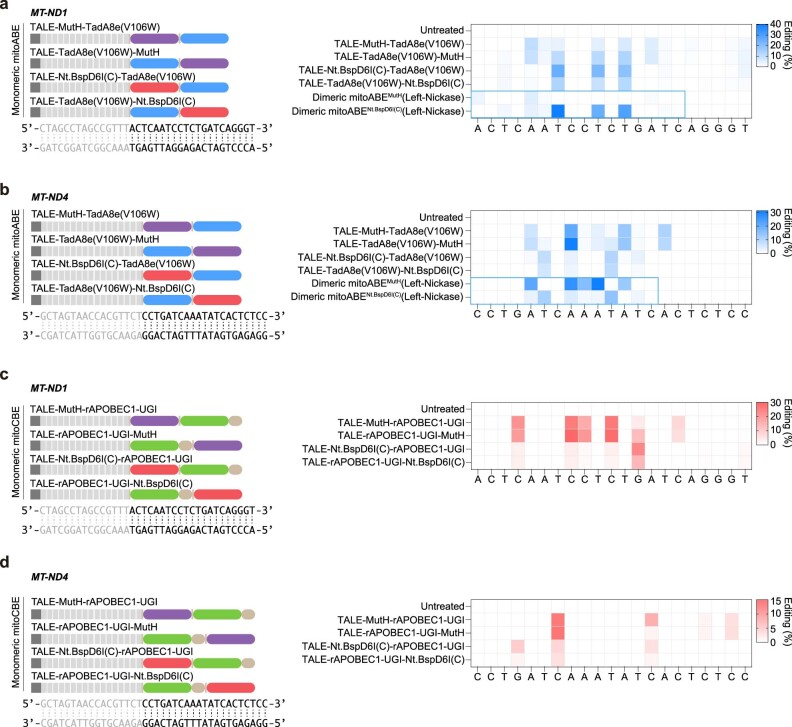
Monomeric mitoBEs enable editing on targeted sites. **a** and **b**, The editing efficiency of monomeric mitoABEs, including TALE-MutH-TadA8e(V106W), TALE-TadA8e(V106W)-MutH, and TALE-Nt.BspD6I(C)-TadA8e(V106W), TALE-TadA8e(V106W)-Nt.BspD6I(C) at *MT-ND1* (**a**) and *MT-ND4* (**b**). The mean values from n = 3 biologically independent replicates are shown.In **a** and **b**, the blue box represents the editing window of the dimeric mitoABEs. **c** and **d**, The editing efficiencies of monomeric mitoCBEs, including TALE-MutH-rAPOBEC1-UGI, TALE-rAPOBEC1-UGI-MutH, and TALE-Nt.BspD6I(C)-rAPOBEC1-UGI, TALE-rAPOBEC1-UGI-Nt.BspD6I(C) at *MT-ND1* (**c**) and *MT-ND4* (**d**). The mean values from n = 3 biologically independent replicates are shown. In **a**-**d**, the purple rounded rectangle represents MutH, the red rounded rectangle represents Nt.BspD6I(C), the gray rectangle represents TALE, the blue rounded rectangle represents TadA8e(V106W), the green rounded rectangle represents rAPOBEC1 and the brown rounded rectangle represents UGI.

Although it is beneficial to have nickase and deaminase domains in two separate TALE arrays, it is tempting to test if they could still work when fused with the same TALE array. We constructed four versions of such mitoABEs: TALE–MutH–TadA8e-V106W, TALE–TadA8e-V106W–MutH, TALE–Nt.BspD6I(C)–TadA8e-V106W and TALE–TadA8e-V106W–Nt.BspD6I(C). Monomeric mitoABEs, mitoABE^MutH^ and mitoABE^Nt.BspD6I(C)^ enabled efficient A-to-G editing (Extended Data Fig. 5a,b). The monomeric versions of mitoABE^MutH^ achieved higher editing efficiency at the *MT-ND1* target site compared with dimeric mitoABE^MutH^ (left MutH), whereas the dimeric mitoABE^Nt.BspD6I(C)^ yielded higher editing efficiency. For the *MT-ND4* site, both monomeric and dimeric mitoABEs showed comparable editing efficiency. Moreover, monomeric mitoABEs have a wider editing window compared with dimeric mitoABEs, with a consistent strand preference observed for both types within the editing windows (Extended Data Fig. 5a,b). The smaller size of monomeric mitoBEs makes them easier to deliver, especially when using AAVs (adeno-associated virus) as a vector. In addition, we also successfully constructed monomeric mitoCBEs (mitoCBE^MutH^ and mitoCBE^Nt.BspD6I(C)^) and achieved efficient C-to-T editing at targeted sites (Extended Data Fig. 5c,d).

### Editing specificity of mitoBEs

**Fig. 5:**
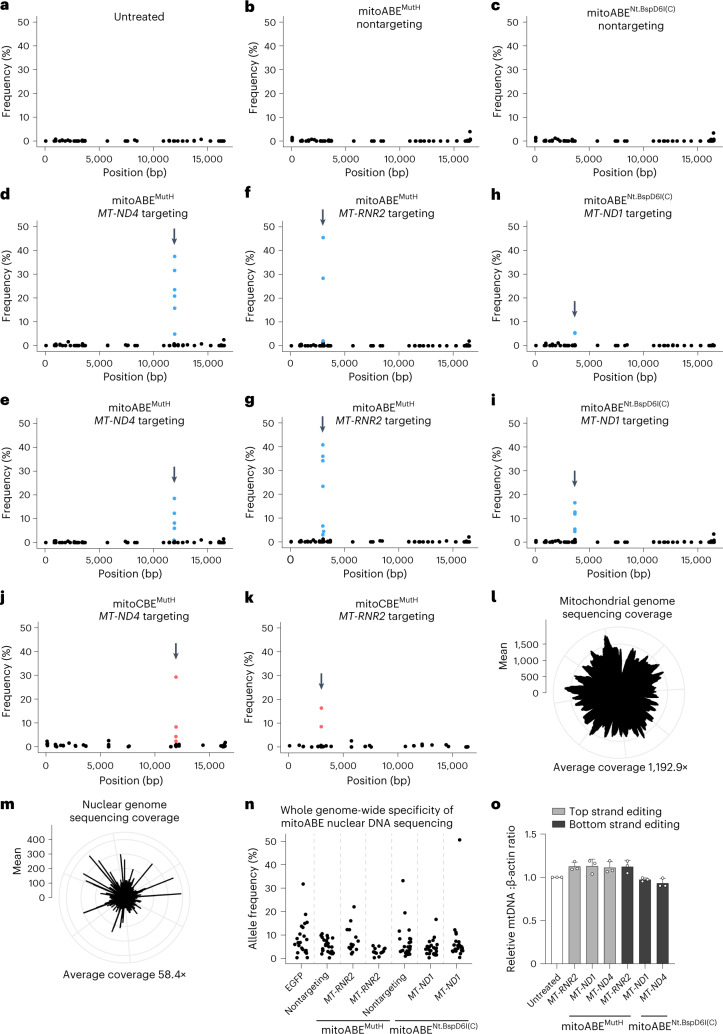
Editing specificity of mitoBEs. **a**–**k**, The average frequency and mitochondrial genome position of each unique single nucleotide variant are shown for untreated HEK293T cells (**a**) and HEK293T cells treated with nontargeting mitoABE^MutH^ (**b**), nontargeting mitoABE^Nt.BspD6I(C)^ (**c**), *MT-ND4*-targeting mitoABE^MutH^ (left TALE–MutH with right TALE–TadA8e-V106W) (**d**), *MT-ND4*-targeting mitoABE^MutH^ (left TALE–TadA8e-V106W with right TALE–MutH) (**e**), *MT-RNR2*-targeting mitoABE^MutH^ (left TALE–MutH with right TALE–TadA8e-V106W) (**f**), *MT-RNR2*-targeting mitoABE^MutH^ (left TALE–TadA8e-V106W with right TALE–MutH) (**g**), *MT-ND1*-targeting mitoABE^Nt.BspD6I(C)^ (left TALE–Nt.BspD6I(C) with right TALE–TadA8e-V106W) (**h**), *MT-ND1*-targeting mitoABE^Nt.BspD6I(C)^ (left TALE–TadA8e-V106W with right TALE–Nt.BspD6I(C)) (**i**), *MT-ND4*-targeting mitoCBE^MutH^ (left TALE–rAPOBEC1–2×UGI with right TALE–MutH) (**j**) and *MT-RNR2*-targeting mitoCBE^MutH^ (left TALE–MutH with right TALE–rAPOBEC1–2×UGI) (**k**). **l**,**m**, The deep sequencing average coverage of the mitochondrial genome (**l**) and nuclear genome (**m**). **n**, The nuclear genome average frequency of each unique single nucleotide variant are shown for the EGFP group (control), nontargeting groups and targeting groups. For **a**–**k** and **n**, all data are three or more biological replicates, the arrow points to the targeted editing site and the blue or red dots represent the editing efficiency of adenines or cytosines in the editing window. For **l** and **m**, all data are presented as mean values of *n* = 3 independent biological replicates. **o**, The copy number of mtDNA was detected by quantitative PCR. Data are presented as mean values ±s.d. of *n* = 3 independent biological replicates.

**Extended Data Fig. 6:**
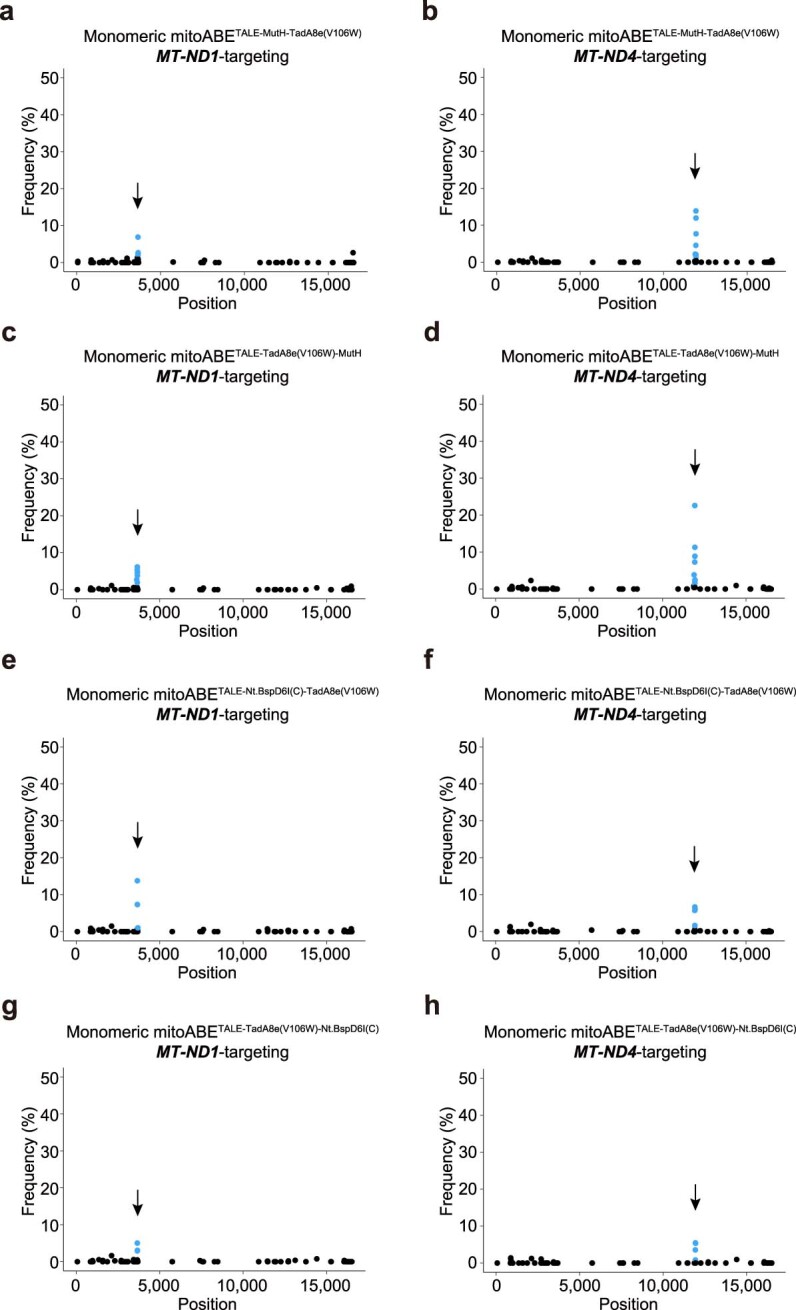
Editing specificity of monomeric mitoABEs. **a-h**, The average frequency and mitochondrial genome position of each unique single nucleotide variant (SNV) are shown for *MT-ND1*-targeting monomeric mitoABE^MutH^ (TALE-MutH-TadA8e(V106W)) (**a**), *MT-ND4*-targeting monomeric mitoABE^MutH^ (TALE-MutH-TadA8e(V106W)) (**b**), *MT-ND1*-targeting monomeric mitoABE^MutH^ (TALE-TadA8e(V106W)-MutH) (**c**), *MT-ND4*-targeting monomeric mitoABE^MutH^ (TALE-TadA8e(V106W)-MutH) (**d**), *MT-ND1*-targeting monomeric mitoABE^Nt.BspD6I(C)^ (TALE-Nt.BspD6I(C)-TadA8e(V106W)) (**e**), *MT-ND4*-targeting monomeric mitoABE^Nt.BspD6I(C)^ (TALE-Nt.BspD6I(C)-TadA8e(V106W)) (**f**), *MT-ND1*-targeting monomeric mitoABE^Nt.BspD6I(C)^ (TALE-TadA8e(V106W)-Nt.BspD6I(C)) (**g**), *MT-ND4*-targeting monomeric mitoABE^Nt.BspD6I(C)^ (TALE-TadA8e(V106W)-Nt.BspD6I(C)) (**h**). **a**-**h**, all data presented as mean values of n = 3 independent biological replicates. The arrow points to the targeted editing site and the blue dots represent the editing efficiency of adenines in the editing window.

To evaluate the editing specificity of mitoBEs, we performed mtDNA sequencing analysis. HEK293T cells transfected with either mitoABE^MutH^- or mitoABE^Nt.BspD6I(C)^-expressing plasmids were subjected to mtDNA sequencing analysis, in which the untreated group (Fig. 5a) and nontargeting groups, including mitoABE^MutH^ and mitoABE^Nt.BspD6I(C)^ not associated with the TALE array (Fig. 5b,c), were used as a control. The mean sequencing coverage across the mitochondrial genome was approximately 1193× (Fig. 5l). mtDNA sequencing analysis detected no nonspecific editing in all experimental groups compared with controls (untreated and nontargeting); only on-target editing was observed (Fig. 5a–i). Of note, the fact that there was no difference between the nontargeting (Fig. 5b,c) and untreated groups (Fig. 5a) suggests that the free form of either TALE–deaminase or TALE–nickase does not cause any unwanted off-target effects. We also assessed the editing specificity of monomeric mitoABEs (monomeric mitoABE^MutH^ and mitoABE^Nt.BspD6I(C)^) and found their specificity to be comparable with that of dimeric mitoABEs (Extended Data Fig. 6a–h). This suggests that both monomeric and dimeric mitoABEs display high specificity when editing the mitochondrial genome. Additionally, we compared the off-target editing of mitoCBEs to that of DdCBEs with the same TALE array and found that mitoCBEs induced lower off-target editing in the mitochondrial genome, particularly at the *MT-ND4*-targeted site (Fig. 5j,k and Supplementary Fig. 2a,b). These results demonstrated that mitoBEs represent a reliable set of mitochondrial editing tools with minimal off-target editing on mtDNA.

Mitochondrial gene editing tools such as DdCBEs are known to cause off-target effects in the nuclear genome^13^. To investigate whether mitoBEs also have off-target effects in the nucleus, we performed whole-genome sequencing (with an average coverage of ~58.4×) and compared the overall off-target editing in the targeting group (including mitoABE^MutH^ and mitoABE^Nt.BspD6I(C)^) with that of the EGFP and nontargeting control groups. We found no significant difference between the targeted groups and the control groups (Fig. 5m,n). Furthermore, we analyzed the presence of TALE-dependent off-target effects using the whole-genome sequencing data and found no off-target editing within ±50 bp of the TALE-array binding sequence (including zero or one mismatch) in the nuclear genome (Supplementary Table 1). These findings suggest that mitoBEs exhibit low off-target effects in the nuclear genome. Due to the limitations of current off-target assay methods, more precise approaches will be necessary to assess off-target editing of the nuclear genome in the future.

**Extended Data Fig. 7:**
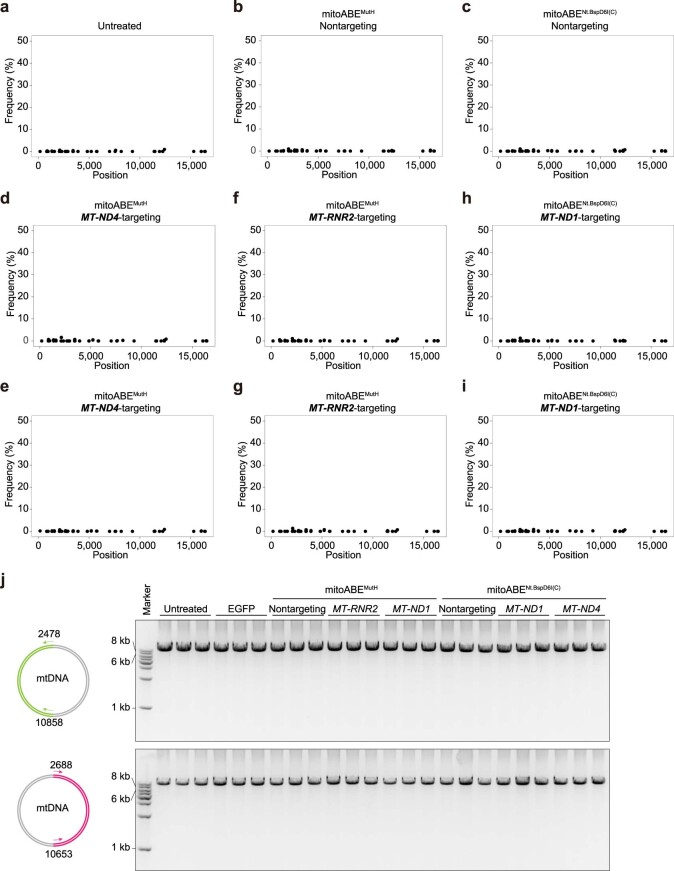
Detection of mitochondrial genome integrity. **a**-**i**, The analysis of indels using high throughput sequencing data. Sample information corresponds to Fig. 6a–i. The mean values from n = 3 biologically independent replicates are shown. **j**, The indels of mitochondrial DNA were detected by long range PCR. See the PCR primers in Supplementary Table 3. Three independent experiments were performed in Extended Data Fig. 7j with similar results.

To further evaluate the effect of mitoABEs on mitochondria, we measured the copy number and integrity of mtDNA. Using the above mtDNA sequencing data, we examined the indels of mtDNA. We found no difference between the targeted groups (Extended Data Fig. 7d–i) and the controls (Extended Data Fig. 7a–c). By real-time quantitative PCR and long-range PCR analysis, we further confirmed that the copy number and integrity of mtDNA in the targeted groups remained the same as those in the controls (Fig. 5o and Extended Data Fig. 7j). Collectively, mitoABEs showed high specificity in human cells.

### Circular RNA-encoded mitoABEs enable strand-biased editing in multiple cell lines

**Fig. 6:**
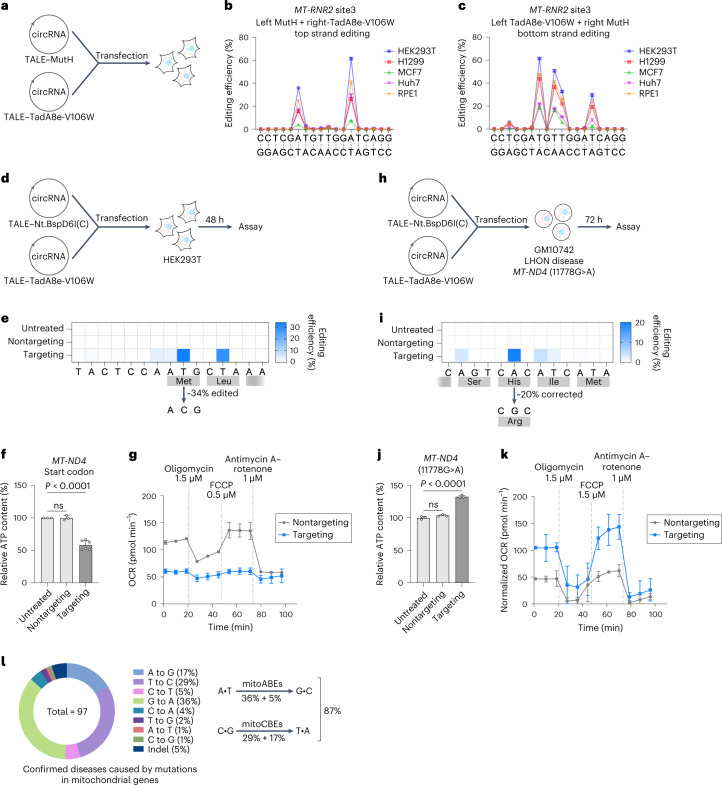
circRNA-encoded mitoABEs successfully created disease models and corrected mutation in cells derived from individuals with LHON. **a**, Overview of circRNA-encoded mitoABE^MutH^-transfected cells. **b**,**c**, circRNAs of two mitoABE^MutH^ orientations were transfected into different cell lines to achieve strand-biased editing, left TALE–MutH with right TALE–TadA8e-V106W (**b**), left TALE–TadA8e-V106W with right TALE–MutH (**c**). Genomic DNA was collected 2 days posttransfection. **d**, Overview of circRNA-encoded mitoABE^Nt.BspD6I(C)^-transfected HEK293T cells and genomic DNA collected 2 days posttransfection. **e**, The editing efficiencies of circRNA-encoded mitoABE^Nt.BspD6I(C)^ targeted the start codon of *MT-ND4*. **f**, The ATP levels of cells transfected with circRNA-encoded mitoABE^Nt.BspD6I(C)^ targeted the start codon of *MT-ND4*. Student’s *t* test, *P* = 2.71 × 10^−5^. **g**, Oxygen consumption rate (OCR) in HEK293T cells treated with circRNA-encoded mitoABE^Nt.BspD6I(C)^ targeted the start codon of *MT-ND4* for 2 days. **h**, Overview of circRNA-encoded mitoABE^Nt.BspD6I(C)^-transfected LHON disease cells GM10742, with genomic DNA was collected 3 days posttransfection. **i**, The editing efficiency of mitoABE^Nt.BspD6I(C)^ corrected the 11778G>A mutation of LHON disease cell GM10742. **j**, The ATP levels of cells transfected with circRNA-encoded mitoABE^Nt.BspD6I(C)^ targeting the 11778G>A mutation of LHON disease cell GM10742. Student’s *t* test, *P* = 6.98 × 10^−5^. **k**, OCR of the LHON disease cell line GM10742 treated with circRNA-encoded mitoABE^Nt.BspD6I(C)^ targeting the 11778G>A mutation for 2 days. **l**, Types of mitochondrial diseases (MITOMAP) and the proportion of diseases that can theoretically be treated by mitoBEs. For **b**, **c**, **f**, **g**, **j** and **k**, the data are presented as mean values ±s.d. of *n* ≥ 3 independent biological replicates. For **e** and **i**, the mean values from *n* = 3 biologically independent replicates are shown. For **g**,**k**, FCCP represents carbonyl cyanide-4 (trifluoromethoxy) phenylhydrazone.

Treatment of disease by direct delivery of RNA shows good potential. Because mitoABEs, unlike the CRISPR system, do not require RNA components to function, we tested mitochondrial editing using circular RNA (circRNA)^34^ to encode mitoABEs. circRNA-encoded mitoABEs conferred strand-biased editing in various human cell types, including H1299, MCF7, Huh7 and RPE1, indicating mitoABEs are versatile tools compatible with various delivery routes to achieve efficient and precise mtDNA base editing (Fig. 6a–c).

### Editing start codons of mitochondrial genes perturbed the function of the respiratory chain

**Extended Data Fig. 8:**
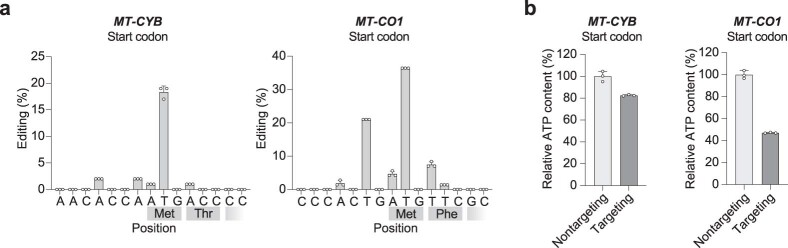
mitoABE^Nt.BspD6I(C)^ edited the start codons of mitochondrial genes to affect mitochondrial functions. **a**, The editing efficiencies of circRNA-encoded mitoABE^Nt.BspD6I(C)^ targeted the start codons of *MT-CYB* and *MT-CO1*. **b**, The ATP levels of cells transfected with circRNA-encoded mitoABE^Nt.BspD6I(C)^ targeted the start codons of *MT-CYB* and *MT-CO1*. For **a** and **b**, data are presented as mean values ± s.d. of n = 3 independent biological replicates.

Mitochondrial diseases are genetic disorders caused by mutations in either the nuclear DNA or mtDNA that are characterized by defects in oxidative phosphorylation^35^. Approximately 90% of mitochondrial genetic disorders caused by mtDNA mutations are due to single base mutations of the mitochondrial coding genes^36^. The leading cause of these genetic disorders is a decrease in ATP production due to the defective assembly of the mitochondrial respiratory complex^37^. Using circRNA-encoded mitoABEs to target the start codons of three genes in HEK293T cells, we tested our editing tools to generate phenotypes mimicking real mitochondrial diseases (Fig. 6d). We chose to target *MT-ND4*, *MT-CYB* and *MT-CO1*, which encode proteins that are components of mitochondrial complex I, mitochondrial complex III and mitochondrial complex IV, respectively^38^. Effective editing by mitoABEs altered all ATG start codons at these three loci by changing T (actually edited A on the noncoding strand) to C, with editing efficiencies of 34%, 18% and 36%, respectively (Fig. 6e and Extended Data Fig. 8a). By measuring the level of intracellular ATP content, editing at all three loci resulted in a decrease in intracellular ATP content (Fig. 6f and Extended Data Fig. 8b). In addition, the cells with the edited start codon of *MT-ND4* exhibited a low rate of respiration oxygen consumption (Fig. 6g). Collectively, these results demonstrated that mitoABEs could edit DNA effectively to create mitochondrial disease models with oxidative respiratory defects.

### Correcting mitochondrial pathogenic DNA mutation via mitoABE

Leber hereditary optic neuropathy (LHON) is the most common inherited mitochondrial disease that affects young adults, and it ultimately leads to acute or subacute blindness^39^. LHON is usually caused by one of three pathogenic mtDNA point mutations. These mutations are located at nucleotide positions 11778G>A, 3460G>A and 14484T>C in the respective *MT-ND4*, *MT-ND1* and *MT-ND6* subunit genes of the mitochondrial oxidative respiratory chain complex I^40,41^. The 11778G>A mutation located at *MT-ND4* changes the highly conserved arginine to histidine (R340H), which accounts for 50% of LHON cases among affected Caucasians people and over 90% of the cases in affected Asian continent individuals^41^. Using circRNA-encoded mitoABE to target GM10742 cells derived from individuals with LHON^42^, we detected a repair efficiency of 20% on a pathogenic mutation (G11778A) (Fig. 6h,i). Importantly, this correction through mitoABE resulted in a significant increase in ATP content and rate of respiration oxygen consumption in GM10742 cells (Fig. 6j,k). Our results demonstrate the strong therapeutic potential of mitoABEs in treating LHON and possibly many other mitochondrial genetic disorders caused by single nucleotide polymorphisms. Currently, 97 mtDNA mutations have been linked to human diseases, with the majority being point mutations (MITOMAP). Of these, 46% are attributed to A•T to G•C mutations, whereas 41% are caused by C•G to T•A mutations. Theoretically, mitoBEs have the potential to model or correct these disease-associated mutations (Fig. 6l).

## Discussion

Mitochondrial base editing techniques are relatively new editing tools that could make specific base substitutions of mtDNA without causing the double-strand breaks that could cause rapid degradation of mtDNA^43^. The realization of targeted mitochondrial base substitutions could greatly empower researchers to study the effects of specific mtDNA mutations and correct disease-causing point mutations for therapeutic purposes. Based on toxin DddA, an enzyme that deaminates cytosine on dsDNA, Mok et al.^10^ developed DdCBE tools that enable programmable C-to-T conversions in mtDNA. Except for DddA, the deaminases found thus far have all been identified as ssDNA deaminases, which cannot deaminate bases on dsDNA. We presumed nickases could nick dsDNA and subsequently induce the ssDNA structure. In this study, we tested the idea of using deaminase activity by generating single strands on target loci using TALE-fused nickase. By combining TALE–nickase and TALE–deaminase, we develop mitochondrial base editing tools, named mitoBEs. Both the A-to-G and C-to-T base conversion, designated mitoABEs and mitoCBEs, respectively, can be achieved using the same strategy.

Among all candidate nickases, MutH could be used in mitoBEs. We were able to generate mutant MutH, MutH*, which requires only the presence of 5′-GAT-3′ (instead of the original 5′-GATC-3′ sequence) to activate its nicking activity, greatly expanding the scope of mitoBE^MutH^ for mtDNA editing (Fig. 2). In addition, we identified Nt.BspD6I(C) as a suitable nickase that does not have any recognition sequence restrictions, thus expanding the range of targets for mitoBEs (Fig. 3). Although TALE–Nt.BspD6I(C) showed reduced strand preference compared with TALE–MutH, it had a wider targeting range. If more precise editing of a specific DNA strand is necessary, we suggest using mitoBE^MutH^ or mitoBE^MutH*^.

mitoBE^MutH^ editing efficiency may be affected by the methylation state of mtDNA because the wild-type MutH can only nick unmethylated and hemimethylated DNA, whereas the F94A variant can nick all three methylation states^20^. In addition, there is a concern about whether nickases may cause double-strand breaks in DNA. However, the TALE–MutH does not induce double-strand breaks in vitro^21^, and we did not observe any mitochondrial genome copy number variations or indels, suggesting that nickases are unlikely to cause double-strand breaks in mtDNA.

Interestingly, we found that the distance of TALE binding and MutH nick motif (5′-GATC-3′) determined the strand for nicking, consequently determining strand-selective editing. mitoABEs and mitoCBEs could enable targeted editing in the human mitochondrial genome, offering powerful tools to either generate mitochondrial disease models or correct most mitochondrial pathogenic point mutations (Fig. 6). Unlike DdCBEs, which induce substantial off-target editing in both nuclear and mitochondrial genomes due to spontaneous assembly of the split DddA halves and the interaction of DddA with CTCF^13^, mitoBEs exhibit much lower off-target risk. mitoBEs may have a better specificity profile because the deaminases used in mitoBEs exhibit high deamination activity only on ssDNA, with low or even no activity on dsDNA; the specificity of dimeric mitoBEs depends on both TALEs binding to their recognition sites, and either TALE–deaminase or TALE–nickase alone is unable to generate base editing; and mitoBEs exhibit strong strand selection for editing. In addition, the editing window of dimeric mitoBEs is controllable because it is determined by two TALE binding sequences. As a result, we generally recommend the use of dimeric mitoBEs. However, delivery of these tools can be challenging due to the packaging restrictions associated with AAVs. In such cases, the use of monomeric mitoBEs is advantageous.

Furthermore, we expanded the cytosine deaminases beyond DddA for mitochondrial C-to-T base editing. Notably, other cytosine deaminases, including AID, A3A, evoAPO, evoFERNY and evoCDA^1^, are all worthy of being tested for their compatibility with our system to remove the restriction of the inherent sequence preference by DddA^10,44^.

In conclusion, mitoBEs are effective and precise base editing tools with broad applicability for editing the mitochondrial genome. We anticipate such tools to be broadly used in basic research and therapeutics in treating diseases associated with mitochondrial defects.

## Methods

### Plasmid construction

PCR was performed using PrimeSTAR GXL DNA Polymerase (TaKaRa) or Q5 Hot Start High-Fidelity DNA Polymerase (NEB). Wild-type TadA, TadA8e-V106W, MutH and its variants, Nt.BspD6I and other genes were synthesized as gene blocks and codon optimized for mammalian expression (Tsingke Biological Technology). The corresponding sequences are listed in the Supplementary Information. We constructed the original mitoBE expression plasmids (the TALE array was replaced with two inverted BsmBI restriction sites) into the pCMV vector by Gibson assembly using Gibson Assembly Master Mix (NEB) and then assembled the TALE array through the Advanced ULtiMATE System^45–47^ (see Supplementary Table 2 for all TALE-array recognition sequences in this manuscript). Ligated plasmids were transformed into Trans1-T1 chemically competent cells (TransGene Biotech) and subjected to Sanger sequencing to analyze the identity of the constructs (Tsingke Biological Technology). Final plasmids were prepared (TianGen) for cell transfection.

### circRNA preparation

circRNAs were prepared according to previous reports^34^. Briefly, the precursor circRNAs were synthesized from the linearized circRNA plasmid templates via in vitro transcription with the HiScribe T7 High Yield RNA Synthesis Kit (NEB), and the reaction products were treated with DNase I (NEB) for 30 min to digest the plasmid templates. After DNase I digestion, GTP was added to the reaction at a final concentration of 2 mM and incubated at 55 °C for 15 min to catalyze the cyclization of circRNAs. Then, the RNA was purified with the Monarch RNA Cleanup Kit (NEB). The purified RNA was heated at 65 °C for 3 min and cooled on ice. The reactions were treated with RNase R (Epicenter) at 37 °C for about 15–30 min to further enrich the circRNAs. The RNase R-treated RNA was purified again with the Monarch RNA Cleanup Kit.

### Cell culture and transfection

HEK293T (CRL-3216; ATCC), NCI-H1299 (CRL-5803; ATCC), MCF7 (HTB-22; ATCC), Huh-7 (JCRB0403; JCRB) and RPE-1 (CRL-4000; ATCC) cells were cultured in DMEM (Gibco) with 10% fetal bovine serum (Biological Industries), 1% GlutaMax (Gibco) and penicillin–streptomycin (Sigma) at 37 °C with 5% CO_2_. GM10742 cells (Coriell Institute) were cultured in RPMI 1640 (Gibco) with 10% fetal bovine serum (Biological Industries), 1% GlutaMax (Gibco) and penicillin–streptomycin (Sigma) at 37 °C with 5% CO_2_. For lipofection, cells were plated in 12-well cell culture plates at a density that reached approximately 70% after 20 h. Cells in each well were transfected with 2,000 ng of each mitoBE monomer using 8 μl of PEI (polyethyleneimine) (ProteinTech) or transfected with 2,500 ng of each mitoBE monomer circRNA using 5 μl of Lipofectamine MessengerMAX Reagent (Invitrogen). Cells were collected after 72 h of transfection. Genomic DNA was extracted using the DNeasy Blood and Tissue Kit (Qiagen) and stored at –20 °C.

### Nucleofection

For GM10742 suspension cells, the circRNA that encoded mitoABE was nucleofected. Briefly, 2 × 10^6^ GM10742 cells were collected and washed with DPBS (Dulbecco’s phosphate-buffered saline). Then, 5,000 ng of each mitoBE monomer circRNA was added to 18 μl of nucleofector supplement and 82 μl of nucleofector solution mix (Lonza). The GM10742 cells were resuspended in the above mix and transferred into a nucleocuvette strip. Then, the nucleocuvette strip was placed into the retainer of the 4D-Nucleofector (Lonza), and nucleofection was initiated with the program DN-100.

### Targeted deep sequencing

Genomic sites of interest were amplified into fragments of approximately 200 bp from genomic DNA samples using PrimeSTAR GXL DNA Polymerase (TaKaRa). See Supplementary Table 3 for the list of primers used and the average mapped ratio of corresponding primers. PCR products were purified using DNA Clean & Concentrator-25 (Zymo Research) for Sanger sequencing and targeted deep sequencing. Targeted deep sequencing libraries were prepared using the VAHTS Universal DNA Library Prep Kit for Illumina v.3 (Vazyme). Briefly, the PCR fragments were sequentially subjected to end repair, adapter ligation and then PCR amplification. DNA purification in library preparation was performed using Agencourt Ampure XP beads (Beckman Coulter), and library amplification was performed using Q5U Hot Start High-Fidelity DNA Polymerase (NEB) and VAHTS Multiplex Oligos Set 4/5 for Illumina (Vazyme). The final library was subjected to quantification using the Qubit dsDNA HS Assay Kit (Invitrogen) and sequenced using Illumina HiSeq X Ten.

### Genome-wide off-target sequencing

We input 500–1,000 ng of genomic DNA for library preparation using the VAHTS Universal Plus DNA Library Prep Kit for Illumina (Vazyme). The library preparation process was as follows: fragmentation, end preparation and dA tailing, adapter ligation and library amplification. A mass of 500–1,000 ng of genomic DNA was fragmented with FEA (Fragmentation, End Preparation & dA-Tailing) enzyme mix at 37 °C for 10 min, and end repair and dA-tailing were simultaneously completed in the process. The final library was subjected to quantification using the Qubit dsDNA HS Assay Kit (Invitrogen) and fragment analyzer. All libraries were finally sequenced using Illumina HiSeq X Ten (Illumina).

### Analysis of high-throughput sequencing data for targeted amplicon sequencing

For high-throughput sequencing data analysis, an index was generated using the targeted site sequences (upstream and downstream ~100 nucleotides) of editing window-covered regions. The reads were aligned and quantified using BWA (v.0.7.10-r789). The BAM alignment files were then sorted with SAMtools (v.1.1), and the editing sites were analyzed using REDitools (v.1.0.4)^48^. The parameters were as follows: -t 8, -U [AG], -n 0.0, -T 6-6, -e, -d, and -u. All the significant base conversions within the targeted regions calculated by Fisher’s exact test (*P* < 0.05) were considered edits made by the mitoBE. The mutations that appeared in the control and experimental groups simultaneously were considered to be due to single nucleotide polymorphisms.

### Analysis of mitochondrial genome off-target editing

The quality control of whole-genome sequencing was conducted using FastQC (v.0.11.9), and adapters were removed by fastp (v.0.20.1). After trimming, reads were mapped to GRCh38-hg38 by bwa-mem2 (v.2.2.1) with default parameters. GATK (v.4.3.0.0)^49^ MergeBamAlignment, MarkDuplicates and BaseRecalibrator were used subsequently to add read group, remove duplicates and correct base quantity. After preprocessing, GATK Mutect2 was used to discover somatic short variants. Variant calls were filtered according to FilterMutectCalls (not annotated as position, slippage, weak evidence or map qual). Mutations with a frequency of more than 1% in the control experiments were also removed. In addition, coverage was evaluated by sambamba (v.0.6.6).

### Analysis of nuclear genome off-target editing

To obtain potential nuclear genome off-target editing events, we used more stringent criteria due to high noise. We added requirements for base quality and mapping quality on the basis of the quality control criteria for mitochondria. Only mutations with a high median base quality (≥30) and high mapping quality (≥50) were considered to be potential off-target editing sites. The Mann–Whitney *U-*test (*P* < 0.1) was used to test whether there was a significant difference between the mutation frequency of each experimental group and the control group. Bowtie2 (v.2.4.5) was used to search similar TALE sequences in human genome, with parameters set to -L 3, -p 4, -D 20, -R 3, and -a. Bedtools (v.2.30.0) was used to check whether there were overlaps between similar TALE sequences and single nucleotide variants found by GATK. When calculating the coverage, we set the scratch window size to 100,000. A few extremely high values were discarded.

### ATP content analysis

ATP content was measured using a firefly luciferase-based ATP assay kit (Beyotime) according to the manufacturer’s instructions. In brief, the cells transfected in 12-well plates for 3 days were lysed using 100 μl of lysis buffer per well and centrifuged at 12,000*g* for 5 min at 4 °C, and the supernatants were removed for the detection of ATP. Then, 20 μl of supernatant was mixed immediately with 100 μl of dilution buffer containing luciferase, which was preincubated at room temperature for 3 min. Relative luminescence units were determined by using a Luminometer (Tecan). The concentration of ATP was calculated according to the standard curve and normalized using the cellular protein level.

### Oxygen consumption analysis

The oxygen consumption rate of cells was measured using an Agilent Seahorse XF Cell Mito Stress Test Kit (Agilent Technologies) according to the manufacturer’s instructions. HEK293T cells (5 × 10^4^ cells per well) were seeded in the Seahorse XF Cell Culture Microplate using the appropriate cell culture growth medium for 24 h before analysis in the Seahorse XFe24 Analyzer (Agilent Technologies). Analysis was performed in Seahorse XF DMEM pH 7.4 (Agilent) with 10 mM glucose (Agilent), 2 mM l-glutamine (Gibco) and 1 mM sodium pyruvate (Gibco). The mitochondrial function of the cells was analyzed by sequential injections of modulators (final concentration of 1.5 μM oligomycin, 0.5 μM FCCP (carbonyl cyanide-4(trifluoromethoxy) phenylhydrazone) and 1 μM antimycin A–rotenone).

GM10742 cells (1.5 × 10^5^ cells per well) were seeded in a polylysine-coated Seahorse XF Cell Culture Microplate using the appropriate cell culture growth medium before analysis in a Seahorse XFe24 Analyzer (Agilent Technologies). Analysis was performed in Seahorse XF RPMI 1640 pH 7.4 (Agilent) with 10 mM glucose (Agilent), 2 mM l-glutamine (Gibco) and 1 mM sodium pyruvate (Gibco). The mitochondrial function of the cells was analyzed by sequential injections of modulators (final concentration of 1.5 μM oligomycin, 1.5 μM FCCP, and 1 μM antimycin A–rotenone).

### Determination of relative total mtDNA levels by quantitative PCR

Quantitative PCR reactions were performed on a LightCycler 96 Instrument (Roche) using SYBR Green (TaKaRa). A mass of 10 ng of purified genomic DNA was used as template input in a 20 μl reaction volume. The level of mtDNA was determined by calculating the ratio of total mtDNA to genomic DNA (β-actin). See Supplementary Table 3 for the list of primers used.

### Long-range PCR to detect mtDNA deletions

Long-range PCR was performed on purified genomic DNA to capture the whole mtDNA genome as two overlapping fragments of approximately 8 kb each. All 100 ng of purified genomic DNA was amplified using the primers (fwd_2478-10858, rev_2478-10858, fwd_2688-10653 and rev_2688-10653) listed in Supplementary Table 3 and PrimeSTAR GXL DNA Polymerase (TaKaRa) in a total reaction volume of 50 μl using the following protocol: 98 °C for 3 min and then 30 cycles of 98 °C for 30 s, 60 °C for 15 s, 68 °C for 8 min and a final 68 °C extension for 5 min. Unpurified PCR products were run on a 1% agarose gel and stained with ethidium bromide. Final imaging was performed with a ChemiDoc Imaging System (Bio-Rad).

### Statistics and reproducibility

*n* represents the number of independent experiments performed in parallel. Unpaired, two-tailed Student’s *t* tests were used for group comparisons as indicated in the figure legends. Significance was classified as follows: *P < 0.05, **P < 0.01, ***P < 0.001, ****P < 0.0001. Three independent experiments were performed in Extended Data Fig. 7j with similar results. For off-target analysis, three independent experiments were performed for the targeted or nontargeted group and seven independent experiments were performed for the untreated group.

### Reporting summary

Further information on research design is available in the Nature Portfolio Reporting Summary linked to this article.

## Online content

Any methods, additional references, Nature Portfolio reporting summaries, source data, extended data, supplementary information, acknowledgements, peer review information; details of author contributions and competing interests; and statements of data and code availability are available at 10.1038/s41587-023-01791-y.

## Supplementary information

Supplementary Information Supplementary Figs. 1 and 2, mitoBE protein sequences and Tables 1–3.

Reporting Summary

Supplementary Tables Supplementary Table 1. TALE-dependent off-target sequence. Supplementary Table 2. TALE array sequence. Supplementary Table 3. Primer sequence.

## Data Availability

All data and materials presented in this manuscript are available from the corresponding author (W.W.) upon reasonable request. Raw data of off-target analysis are available as a BioProject with project identifier PRJCA016204 in the China National Center for Bioinformation–National Genomics Data Center database^50^. The crystal structure of MutH interacting with unmethylated 5′-GATC-3′ is available in the Protein Data Bank database (PDB: 2AOQ). The confirmed human disease-related mtDNA mutations in Fig. 6l are calculated from the MITOMAP database.

## References

[1] Anzalone, A. V., Koblan, L. W. & Liu, D. R. Genome editing with CRISPR-Cas nucleases, base editors, transposases and prime editors. *Nat. Biotechnol.* **38**, 824–844 (2020). 32572269 10.1038/s41587-020-0561-9

[2] Li, G. et al. Gene editing and its applications in biomedicine. *Sci. China Life Sci.* **65**, 660–700 (2022). 35235150 10.1007/s11427-021-2057-0 PMC8889061

[3] Gammage, P. A., Moraes, C. T. & Minczuk, M. Mitochondrial genome engineering: the revolution may not be CRISPR-ized. *Trends Genet.* **34**, 101–110 (2018). 29179920 10.1016/j.tig.2017.11.001 PMC5783712

[4] Alston, C. L., Rocha, M. C., Lax, N. Z., Turnbull, D. M. & Taylor, R. W. The genetics and pathology of mitochondrial disease. *J. Pathol.* **241**, 236–250 (2017). 27659608 10.1002/path.4809 PMC5215404

[5] Gammage, P. A. et al. Genome editing in mitochondria corrects a pathogenic mtDNA mutation in vivo. *Nat. Med.* **24**, 1691–1695 (2018). 30250142 10.1038/s41591-018-0165-9 PMC6225988

[6] Bacman, S. R. et al. MitoTALEN reduces mutant mtDNA load and restores tRNA(Ala) levels in a mouse model of heteroplasmic mtDNA mutation. *Nat. Med.* **24**, 1696–1700 (2018). 30250143 10.1038/s41591-018-0166-8 PMC6942693

[7] Hashimoto, M. et al. MitoTALEN: a general approach to reduce mutant mtDNA loads and restore oxidative phosphorylation function in mitochondrial diseases. *Mol. Ther.* **23**, 1592–1599 (2015). 26159306 10.1038/mt.2015.126 PMC4817924

[8] Gammage, P. A., Rorbach, J., Vincent, A. I., Rebar, E. J. & Minczuk, M. Mitochondrially targeted ZFNs for selective degradation of pathogenic mitochondrial genomes bearing large-scale deletions or point mutations. *EMBO Mol. Med.* **6**, 458–466 (2014). 24567072 10.1002/emmm.201303672 PMC3992073

[9] Lee, H. et al. Mitochondrial DNA editing in mice with DddA-TALE fusion deaminases. *Nat. Commun.* **12**, 1190 (2021). 33608520 10.1038/s41467-021-21464-1 PMC7895935

[10] Mok, B. Y. et al. A bacterial cytidine deaminase toxin enables CRISPR-free mitochondrial base editing. *Nature* **583**, 631–637 (2020). 32641830 10.1038/s41586-020-2477-4 PMC7381381

[11] Mi, L. et al. DddA homolog search and engineering expand sequence compatibility of mitochondrial base editing. *Nat. Commun.* **14**, 874 (2023). 36797253 10.1038/s41467-023-36600-2 PMC9935910

[12] Cho, S. I. et al. Targeted A-to-G base editing in human mitochondrial DNA with programmable deaminases. *Cell* **185**, 1764–1776 (2022). 35472302 10.1016/j.cell.2022.03.039

[13] Lei, Z. et al. Mitochondrial base editor induces substantial nuclear off-target mutations. *Nature* **606**, 804–811 (2022). 35551512 10.1038/s41586-022-04836-5

[14] Richter, M. F. et al. Phage-assisted evolution of an adenine base editor with improved Cas domain compatibility and activity. *Nat. Biotechnol.* **38**, 883–891 (2020). 32433547 10.1038/s41587-020-0453-z PMC7357821

[15] Wolf, J., Gerber, A. P. & Keller, W. tadA, an essential tRNA-specific adenosine deaminase from Escherichia coli. *EMBO J.* **21**, 3841–3851 (2002). 12110595 10.1093/emboj/cdf362 PMC126108

[16] Mak, A. N., Bradley, P., Cernadas, R. A., Bogdanove, A. J. & Stoddard, B. L. The crystal structure of TAL effector PthXo1 bound to its DNA target. *Science* **335**, 716–719 (2012). 22223736 10.1126/science.1216211 PMC3427646

[17] Deng, D. et al. Structural basis for sequence-specific recognition of DNA by TAL effectors. *Science* **335**, 720–723 (2012). 22223738 10.1126/science.1215670 PMC3586824

[18] Sanjana, N. E. et al. A transcription activator-like effector toolbox for genome engineering. *Nat. Protoc.* **7**, 171–192 (2012). 22222791 10.1038/nprot.2011.431 PMC3684555

[19] Ban, C. & Yang, W. Structural basis for MutH activation in E.coli mismatch repair and relationship of MutH to restriction endonucleases. *EMBO J.* **17**, 1526–1534 (1998). 9482749 10.1093/emboj/17.5.1526 PMC1170500

[20] Friedhoff, P., Thomas, E. & Pingoud, A. Tyr212: a key residue involved in strand discrimination by the DNA mismatch repair endonuclease MutH. *J. Mol. Biol.* **325**, 285–297 (2003). 12488096 10.1016/s0022-2836(02)01224-x

[21] Gabsalilow, L., Schierling, B., Friedhoff, P., Pingoud, A. & Wende, W. Site- and strand-specific nicking of DNA by fusion proteins derived from MutH and I-SceI or TALE repeats. *Nucleic Acids Res.* **41**, e83 (2013). 23408850 10.1093/nar/gkt080 PMC3627573

[22] Lee, J. Y. et al. MutH complexed with hemi- and unmethylated DNAs: coupling base recognition and DNA cleavage. *Mol. Cell* **20**, 155–166 (2005). 16209953 10.1016/j.molcel.2005.08.019

[23] Pingoud, A., Fuxreiter, M., Pingoud, V. & Wende, W. Type II restriction endonucleases: structure and mechanism. *Cell. Mol. Life Sci.* **62**, 685–707 (2005). 15770420 10.1007/s00018-004-4513-1 PMC11924531

[24] Kim, D. E., Chivian, D. & Baker, D. Protein structure prediction and analysis using the Robetta server. *Nucleic Acids Res.* **32**, W526–W531 (2004). 15215442 10.1093/nar/gkh468 PMC441606

[25] Zheleznaya, L. A., Perevyazova, T. A., Alzhanova, D. V. & Matvienko, N. I. Site-specific nickase from bacillus species strain d6. *Biochemistry (Mosc.)* **66**, 989–993 (2001). 11703181 10.1023/a:1012369525809

[26] Ramirez, C. L. et al. Engineered zinc finger nickases induce homology-directed repair with reduced mutagenic effects. *Nucleic Acids Res.* **40**, 5560–5568 (2012). 22373919 10.1093/nar/gks179 PMC3384306

[27] Zhu, Z., Samuelson, J. C., Zhou, J., Dore, A. & Xu, S. Y. Engineering strand-specific DNA nicking enzymes from the type IIS restriction endonucleases BsaI, BsmBI, and BsmAI. *J. Mol. Biol.* **337**, 573–583 (2004). 15019778 10.1016/j.jmb.2004.02.003

[28] Xu, S. Y. et al. Discovery of natural nicking endonucleases Nb.BsrDI and Nb.BtsI and engineering of top-strand nicking variants from BsrDI and BtsI. *Nucleic Acids Res.* **35**, 4608–4618 (2007). 17586812 10.1093/nar/gkm481 PMC1950550

[29] Chan, S. H., Zhu, Z., Van Etten, J. L. & Xu, S. Y. Cloning of CviPII nicking and modification system from chlorella virus NYs-1 and application of Nt.CviPII in random DNA amplification. *Nucleic Acids Res.* **32**, 6187–6199 (2004). 15570069 10.1093/nar/gkh958 PMC535667

[30] Zhang, P. et al. Engineering BspQI nicking enzymes and application of N.BspQI in DNA labeling and production of single-strand DNA. *Protein Expr. Purif.* **69**, 226–234 (2010). 19747545 10.1016/j.pep.2009.09.003 PMC2783397

[31] Xu, Y., Lunnen, K. D. & Kong, H. Engineering a nicking endonuclease N.AlwI by domain swapping. *Proc. Natl Acad. Sci. USA* **98**, 12990–12995 (2001). 11687651 10.1073/pnas.241215698 PMC60812

[32] Kleinstiver, B. P. et al. The I-TevI nuclease and linker domains contribute to the specificity of monomeric TALENs. *G3 (Bethesda)* **4**, 1155–1165 (2014). 24739648 10.1534/g3.114.011445 PMC4065259

[33] Komor, A. C., Kim, Y. B., Packer, M. S., Zuris, J. A. & Liu, D. R. Programmable editing of a target base in genomic DNA without double-stranded DNA cleavage. *Nature* **533**, 420–424 (2016). 27096365 10.1038/nature17946 PMC4873371

[34] Qu, L. et al. Circular RNA vaccines against SARS-CoV-2 and emerging variants. *Cell* **185**, 1728–1744 (2022). 35460644 10.1016/j.cell.2022.03.044 PMC8971115

[35] Herst, P. M., Rowe, M. R., Carson, G. M. & Berridge, M. V. Functional mitochondria in health and disease. *Front Endocrinol. (Lausanne)* **8**, 296 (2017). 29163365 10.3389/fendo.2017.00296 PMC5675848

[36] Taylor, R. W. & Turnbull, D. M. Mitochondrial DNA mutations in human disease. *Nat. Rev. Genet.* **6**, 389–402 (2005). 15861210 10.1038/nrg1606 PMC1762815

[37] Ng, Y. S. & Turnbull, D. M. Mitochondrial disease: genetics and management. *J. Neurol.* **263**, 179–191 (2016). 26315846 10.1007/s00415-015-7884-3 PMC4723631

[38] Dimauro, S. & Davidzon, G. Mitochondrial DNA and disease. *Ann. Med.* **37**, 222–232 (2005). 16019721 10.1080/07853890510007368

[39] Priglinger, C., Klopstock, T., Rudolph, G. & Priglinger, S. G. [Leber’s Hereditary Optic Neuropathy]. *Klin. Monbl. Augenheilkd* **236**, 1271–1282 (2019). 31639883 10.1055/a-0972-1552

[40] Wallace, D. C. et al. Mitochondrial DNA mutation associated with Leber’s hereditary optic neuropathy. *Science* **242**, 1427–1430 (1988). 3201231 10.1126/science.3201231

[41] Sundaramurthy, S. et al. Leber hereditary optic neuropathy-new insights and old challenges. *Graefes Arch. Clin. Exp. Ophthalmol.* **259**, 2461–2472 (2021). 33185731 10.1007/s00417-020-04993-1

[42] Chin, R. M., Panavas, T., Brown, J. M. & Johnson, K. K. Patient-derived lymphoblastoid cell lines harboring mitochondrial DNA mutations as tool for small molecule drug discovery. *BMC Res. Notes* **11**, 205 (2018). 29587845 10.1186/s13104-018-3297-6 PMC5870301

[43] Peeva, V. et al. Linear mitochondrial DNA is rapidly degraded by components of the replication machinery. *Nat. Commun.* **9**, 1727 (2018). 29712893 10.1038/s41467-018-04131-w PMC5928156

[44] Mok, B. Y. et al. CRISPR-free base editors with enhanced activity and expanded targeting scope in mitochondrial and nuclear DNA. *Nat. Biotechnol.* **40**, 1378–1387 (2022). 35379961 10.1038/s41587-022-01256-8 PMC9463067

[45] Yang, J. et al. ULtiMATE system for rapid assembly of customized TAL effectors. *PLoS ONE* **8**, e75649 (2013). 24228087 10.1371/journal.pone.0075649 PMC3815405

[46] Yang, J. et al. Complete decoding of TAL effectors for DNA recognition. *Cell Res*. **24**, 628–631 (2014). 24513857 10.1038/cr.2014.19 PMC4011339

[47] Zhang, Y. et al. Deciphering TAL effectors for 5-methylcytosine and 5-hydroxymethylcytosine recognition. *Nat. Commun.* **8**, 901 (2017). 29026078 10.1038/s41467-017-00860-6 PMC5638953

[48] Picardi, E. & Pesole, G. REDItools: high-throughput RNA editing detection made easy. *Bioinformatics* **29**, 1813–1814 (2013). 23742983 10.1093/bioinformatics/btt287

[49] Van der Auwera, G. A. et al. From FastQ data to high confidence variant calls: the Genome Analysis Toolkit best practices pipeline. *Curr. Protoc. Bioinformatics* **43**, 11.10.11–11.10.33 (2013). 10.1002/0471250953.bi1110s43 PMC4243306 25431634

[50] Yi, Z. et al. Strand-selective mitochondrial DNA base editing of human mitochondrial DNA using MitoBEsStrand. National Genomics Data Center https://ngdc.cncb.ac.cn/bioproject/browse/PRJCA016204 (2023).

