# Supplementary material for: Strand-selective base editing of human mitochondrial DNA using mitoBEs
Source: Nat Biotechnol. 2023 May 22;42(3):498–509. doi: 10.1038/s41587-023-01791-y (PMC10940147; doi:10.1038/s41587-023-01791-y)
Supplement: Supplementary file 1 — Supplementary Figs. 1 and 2, mitoBE protein sequences and Tables 1–3. [file 41587_2023_1791_MOESM1_ESM.pdf]

nature biotechnology

Article https://doi.org/10.1038/s41587-023-01791-y

Strand-selective base editing of human mitochondrial DNA using mitoBEs

In the format provided by the authors and unedited

Supplementary Figure 1. Test the linker sequences between TALE and nickase.

Page 2

Supplementary Figure 2. Mitochondrial genome editing specificity of DdCBEs.

Page 3

Supplementary Sequences. The sequence of TALE-Nickases, TALE-Deaminases and monomeric mitoBEs sequence.

Page 4-11

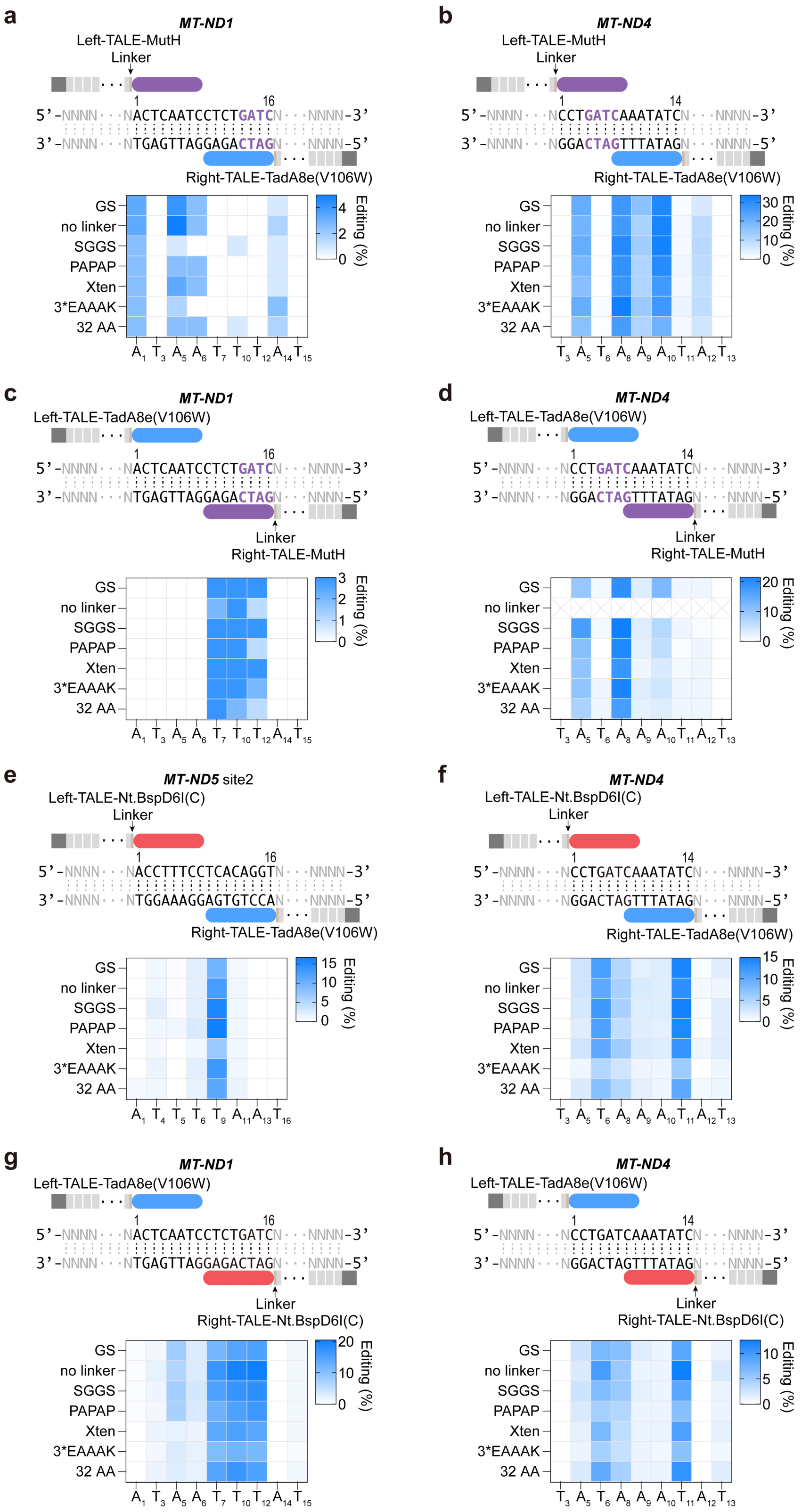

Supplementary Fig. 1 Test the linker sequences between TALE and nickase. a-d, The editing efficiencies of mitoABE^{MutH} with different linkers. **e-h**, The editing efficiencies of mitoABE$^{Nt.BspD6I(C)}$ with different linkers. The mean values from n = 3 biologically independent replicates are shown.

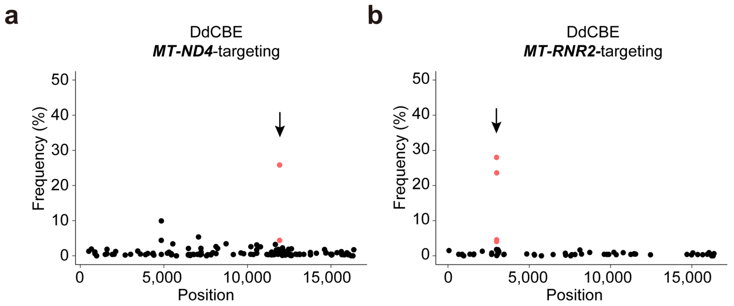

Supplementary Fig. 2 Mitochondrial genome editing specificity of DdCBEs. *MT-ND4*-targeting DdCBE (Left TALE-DddA-G1397-N with Left TALE-DddA-G1397-C) (**a**), *MT-RNR2*-targeting DdCBE (Left TALE-DddA-G1397-N with Left TALE-DddA-G1397-C) (**b**). For **a** and **b**, data are presented as mean values of n = 3 independent biological replicates. The arrow points to the targeted editing site and the red dots represent the editing efficiency of cytosines in the editing window.

Supplementary Sequences.

The sequence of TALE-Nickases, TALE-Deaminases and monomeric mitoBEs sequence.

The TALE repeat array sequences are shown in Supplementary Table. 2

TALE-Effector sequence:

MTS-Tag-TALE N-terminal non-repeat-TALE Repeat array-TALE C-terminal non-repeat-2aa linker-Nickase/Deaminase

Non-Targeting TALE-Effector sequence:

MTS-Tag-TALE N-terminal non-repeat-TALE C-terminal non-repeat-2aa linker-Nickase/Deaminase

MTS:

>SOD2 MTS

MALSRAVCGTSRQLAPVLGYLGSRQKHSLPD

>COX8 MTS

MASVLTPLLLRGLTGSARRLPVPRAK

Tag:

>3×HA

YPYDVPDYAGYPYDVPDYAGYPYDVPDYA

>3×FLAG

IHSLDYKDHDGDYKDHDIDYKDDDDKMDIA

> TALE N-terminal non-repeat

MDIADLRTLGYSQQQQEKIKPKVRSTVAQHHEALVGHGFTHAHIVALSQHPAALGTVAVK
YQDMIAALPEATHEAIVGVGKQWSGARALEALLTVAGELRGPPLQLDTGQLLKIAKRGGVT
AVEAVHAWRNALTGAPLN

>TALE Repeat array (e.g., 20mer)

LTPEQVVAIAS??GGKQALETVQRLLPVLCQAHGLTPEQVVAIAS??GGKQALETVQRLLPVLCQAHGLTPEQVVAIAS??GGKQALETVQRLLPVLCQAHGLTPEQVVAIAS??GGKQALETVQRLLPVLCQAHGLTPEQVVAIAS??GGKQALETVQRLLPVLCQAHGLTPEQVVAIAS??GGKQALETVQRLLPVLCQAHGLTPEQVVAIAS??GGKQALETVQRLLPVLCQAHGLTPEQVVAIAS??GGKQALETVQRLLPVLCQAHGLTPEQVVAIAS??GGKQALETVQRLLPVLCQAHGLTPEQVVAIAS??GGKQALETVQRLLPVLCQAHGLTPEQVVAIAS??GGKQALETVQRLLPVLCQAHGLTPEQVVAIAS??GGKQALETVQRLLPVLCQAHGLTPEQVVAIAS??GGKQALETVQRLLPVLCQAHGLTPEQVVAIAS??GGKQALETVQRLLPVLCQAHGLTPEQVVAIAS??GGKQALETVQRLLPVLCQAHGLTPEQVVAIAS??GGKQALETVQRLLPVLCQAHGLTPEQVVAIAS??GGKQALETVQRLLPVLCQAHGLTPEQVVAIAS??GGKQALETVQRLLPVLCQAHGLTPEQVVAIAS??GGKQALETVQRLLPVLCQAHGLTPEQVVAIAS??GGKQALE

> TALE N-terminal non-repeat

SIVAQLSRPDPALAALTNDHLVALACLGGRPALDAVKKGLG

>2aa linker

GS

>SGGS linker

SGGS

>PAPAP linker

PAPAP

>Xten linker

SGSETPGTSESATPES

>3*EAAAK linker

AEAAAKEAAAKEAAAKEAAAKA

>32AA linker

GSGSSGGSSGSETPGTSESATPESSGGSSGGS

Nickase sequences

>MutH

MSQPRPLLSPPETEEQLLAQAQQLSGYTLGELAALVGLVTPENLKRDKGWIGVLLEIWLGAS
AGSKPEQDFAALGVELKTIPVDSLGRPLETTFVCVAPLTGNSGVTWETSHVRHKLKRVLWIP
VEGERSIPLAQRRVGSPLLWSPNEEEDRQLREDWEELMDMIVLGQVERITARHGEYLQIRPK
AANAKALTEAIGARGERILTLPRGFYLKKNFTSALLARHFLIQ

>Nt.BspD6I(C)

RQLEEVIDLLEVYHEKKNVIEEKIKARFIANKNTVFEWLTWNGFIILGNALEYKNNFVIDEEL
QPVTHAAGNQPDMEIIYEDFIVLGEVTTSKGATQFKMESEPVTRHYLNKKKELEKQGVEKE
LYCLFIAPEINKNTFEEFMKYNIVQNTRIIPLSLKQFNMLLMVQKKLIEKGRRLSSYDIKNLM
VSLYRTTIECERKYTQIKAGLEETLNNWVVDKEVRF

>Fok1-Fok1(D450A)

FKQLVKSELEEKKSELRHKLKYVPHEYIELIEIARNSTQDRILEMKVMEFFMKVYGYRGKHL
GGSRKPDGAIYTVGSPIDYGVIVDTKAYSGGYNLPIGQADEMQRYVEENQTRNKHINPNEW
WKVYPSSVTEFKFLFVSGHFKGNYKAQLTRLNHITNCNGAVLSVEELLIGGEMIKAGTLTLE
EVRRKFNNGEINFSSGGGGSGGGGSGGGGSGGSGGGSSGGGGSGGGGSGGGGSQLVKSELE
EKKSELRHKLKYVPHEYIELIEIARNSTQDRILEMKVMEFFMKVYGYRGKHLGGSRKPAGAI
YTVGSPIDYGVIVDTKAYSGGYNLPIGQADEMQRYVEENQTRNKHINPNEWWKVYPSSVTE
FKFLFVSGHFKGNYKAQLTRLNHITNCNGAVLSVEELLIGGEMIKAGTLTLEEVRRKFNNGE
INF

>Nb.BsaI(C, N441D/R442G)

CRSHADRGRWEKNLRSYTTDRRAFEYWVDGDWVAADKLMGLIRTNEQIKKETCLNDNHP
GPCSADHIGPISLGFVHRPEFQLLCNSCNSAKNNRMTFSDVQHLINAENNGEEVASWYCKHI
WDLRKHDVKNNENALRLSKILRDNRHTAMFILSELLKDNHYLFLSTFLGLQYAERSVSFSNI
KIENHIITGQISEQPRDTKYTEEQKARRMRIGFEALKSYIEKEDGNALLVINDKIIDKINEIKNIL
QDIPDEYKLLNEKISEQFNSEEVSDELLRDLVTHLPTKESEPANFKLARKYLQEIMEIVGDEL
SKMWEDERYVRQTFADLD

>Nt.BsaI(C, R236D)

CRSHADRGRWEKNLRSYTTDRDAFEYWVDGDWVAADKLMGLIRTNEQIKKETCLNDNHP
GPCSADHIGPISLGFVHRPEFQLLCNSCNSAKNNRMTFSDVQHLINAENNGEEVASWYCKHI
WDLRKHDVKNNENALRLSKILRDNRHTAMFILSELLKDNHYLFLSTFLGLQYAERSVSFSNI
KIENHIITGQISEQPRDTKYTEEQKARRMRIGFEALKSYIEKENRNALLVINDKIIDKINEIKNIL
QDIPDEYKLLNEKISEQFNSEEVSDELLRDLVTHLPTKESEPANFKLARKYLQEIMEIVGDEL
SKMWEDERYVRQTFADLD

>Nb.BsmBI (C, R438D)

KDPGRHDDNMRLYNHDRRAFMWWSEGDWALADALYNKAGAGKCADPDCQKEVEKISPD
HVGPISCGFKQIPFFKPLCASCNSAKNRRFSYQDVKELLKYENYTGDSVASWQVRALWDNC
KHLVKNDDDSKLLSNLMRSLQDYYLRSLYKLFSNGFAHLLSYFLTPEYAHYKITFEGLNTST
LEYERYYKTFKKTKSTSSLAARIVRIAFEELEIYNSKDINEDKLIKFDTSSWEKDFENIISYATK
NLSLDEEASKWNKVLTDKNLSSTEKDKKISSLLEDKNYEVYKKQFYILKDLLVEHFNKIGEQ
IAKDYMK

>Nt.BsmAI(C, R221D)

CRASQDKGRSKENLKSYTKDRDAYEYWSDGNIHAANQFMGSPFFNNISADHIGPISLGFVH
DPRYLQPMSGGDNSSKRDRLQLDDIEKIIETEKRTNVYPMSWYSKLIWEYIKKNYSTHKSLIS

GVYRDALKQNMSNFMYILWYILEHCNQDGEHFLEEALLKPNYDYFQYSYTFNELGEIVSIN
PRHFTDRNQYETERYKRIAFESVYDYNEKENRNIKANLIDNEQRMLNKLCQEISSGVPVEQC
KKLLIELMEVIQKRIISTL

>Nb.BsrDI(C)

IPEELFNWPRTDKVNFKSPQGLIKYDELCYQLEKAVGSKKAYCLSNNAGAKPQKLESLKEW
INSQKKLFDKAPKLTPPAEFNMKLDAFPVTSNNNYYVTTSKNILYLFDYWKDLRIAIETAFP
RLKGKLPTDIDEKPALIYICNSVKPGRLFGDPFTGQLSAFSTIFGKKNIDMPRIVVAYYPHQIY
SQALPKNNKSNKGITLKKELTDFLIFHGGVVVKLNEGKAY

>Nt.CviPII

MYIYMSTPQAKTKYYEQRFVNDFYKELERNKVSLPVTIVLKDNLGIKQVIQNGSGVRVLRD
KANAKSPSKIKSEELGRHVTSKADIALFTEEKNGTKVDVAWISPQSHKDFLGKKITPAQYFD
ASSDVMFKTKIGQPKEIKELKNKMISLSVPLTATKYCWPKYKSGTSLRIWDDVQSTILMNM
AIFGVEFGKAYCRNNANILMVGDPLIEVKDDKTIILTTKENGFSLANGFAEYIPSKDKPIFFTK
PTSGKKTVVDGKTIEGVSVWIIYRSYAGSKNRKIDDVLKNKIELISSSCSVKKKDNFVSIMQS
KKITSPPKSKKITSPPKSKKITSPSKSKKITNFFMKK

>BspQI(C)

NNSFNPVRTKDQLHESAVITREKKILLKEPEILQKIKNRNNGEGLKSIIWKKFDKKCFNCEKE
LTIEEVRLDHTRPLAYLWPIDEHATCLCEKCNNTKHDMFPIDFYQGDEDKLRRLARITGLDY
ESLVKRDVNEVELARIINNIEDFATNVEARTFRSIRNKVKEVRPDTDLFEILKSKNINLYNELQ
YELLTRKD

>N.AlwI(C)

YHLEELLFENNEKKFAENQKNEWDEILAYMDLLISPKPISIEIADKEISIPSGERPAYFEWVLW
RAFLALNHLIIEPQQCRRFKVDQDFKPIHNAPGGGADVIFEYENFKILGEVTTSKGATQFKME

SEPVTRHYLNKKKELEKQGVEKELYCLFIAPEINKNTFEEFMKYNIVQNTRIIPLSLKQFNML
LMVQKKLIEKGRRLSSYDIKNLMVSLYRTTIECERKYTQIKAGLEETLNNWVVDKEVRF

>I-TevI
KSGIYQIKNTLNNKVYVGSAKDFEKRWKRHFKDLEKGCHSSIKLQRSFNKHGNVFECSILEE
IPYEKDLIIERENFWIKELNSKINGYNIADATFGDTCSTHPLKEEIIKKRSETVKAKMLKLGPD
GRKALYSKPGSKNGRWNPETHKFCKCGVRIQTSAYTCSKCRNRSGENNSFFNHKHSQGPSA
D

Deaminase sequences
>TadA8e(V106W)
SEVEFSHEYWMRHALTLAKRARDEREVPVGAVLVLNNRVIGEGWNRAIGLHDPTAHAEIM
ALRQGGLVMQNYRLIDATLYVTFEPCVMCAGAMIHSRIGRVVFGWRNSKRGAAGSLMNV
LNYPGMNHRVEITEGILADECAALLCDFYRMPRQVFNAQKKAQSSIN

>rAPOBEC1-2×UGI
SSETGPVAVDPTLRRRIEPHEFEVFFDPRELRKETCLLYEINWGGRHSIWRHTSQNTNKHVE
VNFIEKFTTERYFCPNTRCSITWFLSWSPCGECSRAITEFLSRYPHVTLFIYIARLYHHADPRN
RQGLRDLISSGVTIQIMTEQESGYCWRNFVNYSPSNEAHWPRYPHLWVRLYVLELYCIILGL
PPCLNILRRKQPQLTFFTIALQSCHYQRLPPHILWATGLKGSGGSTNLSDIIEKETGKQLVIQE
SILMLPEEVEEVIGNKPESDILVHTAYDESTDENVMLLTSDAPEYKPWALVIQDSNGENKIK
MLSGGSGGSGGSTNLSDIIEKETGKQLVIQESILMLPEEVEEVIGNKPESDILVHTAYDESTDE
NVMLLTSDAPEYKPWALVIQDSNGENKIKML

Monomeric mitoBE sequence

MTS-Tag-TALE N-terminal non-repeat-TALE Repeat array-TALE C-terminal non-repeat-2aa linker-Nickase-2aa linker-Deaminase

MTS-Tag-TALE N-terminal non-repeat-TALE Repeat array-TALE C-terminal non-repeat-2aa linker-Deaminase -2aa linker-Nickase

>MutH-2aa linker-TadA8e(V106W)

MSQPRPLLSPPETEEQLLAQAQQLSGYTLGELAALVGLVTPENLKRDKGWIGVLLEIWLGASAGSKPEQDFAALGVELKTIPVDSLGRPLETTFVCVAPLTGNSGVTWETSHVRHKLKRVLWIPVEGERSIPLAQRRVGSPLLWSPNEEEDRQLREDWEELMDMIVLGQVERITARHGEYLQIRPKAANAKALTEAIGARGERILTLPRGFYLKKNFTSALLARHFLIQGSSEVEFSHEYWMRHALTLAKRARDEREVPVGAVLVLNNRVIGEGWNRAIGLHDPTAHAEIMALRQGGLVMQNYRLIDATLYVTFEPCVMCAGAMIHSRIGRVVFGWRNSKRGAAGSLMNVLNYPGMNHRVEITEGILADECAALLCDFYRMPRQVFNAQKKAQSSIN

> TadA8e(V106W)-2aa linker- MutH

SEVEFSHEYWMRHALTLAKRARDEREVPVGAVLVLNNRVIGEGWNRAIGLHDPTAHAEIMALRQGGLVMQNYRLIDATLYVTFEPCVMCAGAMIHSRIGRVVFGWRNSKRGAAGSLMNVLNYPGMNHRVEITEGILADECAALLCDFYRMPRQVFNAQKKAQSSINGSMSQPRPLLSPPETEEQLLAQAQQLSGYTLGELAALVGLVTPENLKRDKGWIGVLLEIWLGASAGSKPEQDFAALGVELKTIPVDSLGRPLETTFVCVAPLTGNSGVTWETSHVRHKLKRVLWIPVEGERSIPLAQRRVGSPLLWSPNEEEDRQLREDWEELMDMIVLGQVERITARHGEYLQIRPKAANAKALTEAIGARGERILTLPRGFYLKKNFTSALLARHFLIQ

> Nt.BspD6I(C)-2aa linker-TadA8e(V106W)

RQLEEVIDLLEVYHEKKNVIEEKIKARFIANKNTVFEWLTWNGFIILGNALEYKNNFVIDEELQPVTHAAGNQPDMEIIYEDFIVLGEVTTSKGATQFKMESEPVTRHYLNKKKELEKQGVEKELYCLFIAPEINKNTFEEFMKYNIVQNTRIIPLSLKQFNMLLMVQKKLIEKGRRLSSYDIKNLMVSLYRTTIECERKYTQIKAGLEETLNNWVVDKEVRFGSSEVEFSHEYWMRHALTLAKRARDEREVPVGAVLVLNNRVIGEGWNRAIGLHDPTAHAEIMALRQGGLVMQNYRLIDATLYVTFE

PCVMCAGAMIHSRIGRVVFGWRNSKRGAAGSLMNVLNYPGMNHRVEITEGILADECAALL
CDFYRMPRQVFNAQKKAQSSIN

> TadA8e(V106W)-2aa linker-Nt.BspD6I(C)

SEVEFSHEYWMRHALTLAKRARDEREVPVGAVLVLNNRVIGEGWNRAIGLHDPTAHAEIM
ALRQGGLVMQNYRLIDATLYVTFEPCVMCAGAMIHSRIGRVVFGWRNSKRGAAGSLMNV
LNYPGMNHRVEITEGILADECAALLCDFYRMPRQVFNAQKKAQSSINGSRQLEEVIDLLEVY
HEKKNVIEEKIKARFIANKNTVFEWLTWNGFIILGNALEYKNNFVIDEELQPVTHAAGNQPD
MEIIYEDFIVLGEVTTSKGATQFKMESEPVTRHYLNKKKELEKQGVEKELYCLFIAPEINKNT
FEEFMKYNIVQNTRIIPLSLKQFNMLLMVQKKLIEKGRRLSSYDIKNLMVSLYRTTIECERKY
TQIKAGLEETLNNWVVDKEVRF
