# Supplementary material for: Strand-selective base editing of human mitochondrial DNA using mitoBEs
Source: Nat Biotechnol. 2023 May 22;42(3):498–509. doi: 10.1038/s41587-023-01791-y (PMC10940147; doi:10.1038/s41587-023-01791-y)
Supplement: Supplementary file 2 — Reporting Summary [file 41587_2023_1791_MOESM2_ESM.pdf]

nature portfolio

Corresponding author(s): Wensheng Wei

Last updated by author(s): Apr 8, 2023

Reporting Summary

Nature Portfolio wishes to improve the reproducibility of the work that we publish. This form provides structure for consistency and transparency in reporting. For further information on Nature Portfolio policies, see our Editorial Policies and the Editorial Policy Checklist.

Statistics

For all statistical analyses, confirm that the following items are present in the figure legend, table legend, main text, or Methods section.

n/a	Confirmed	
☐	☒	The exact sample size (n) for each experimental group/condition, given as a discrete number and unit of measurement
☐	☒	A statement on whether measurements were taken from distinct samples or whether the same sample was measured repeatedly
☐	☒	The statistical test(s) used AND whether they are one- or two-sided<br>*Only common tests should be described solely by name; describe more complex techniques in the Methods section.*
☒	☐	A description of all covariates tested
☐	☒	A description of any assumptions or corrections, such as tests of normality and adjustment for multiple comparisons
☐	☒	A full description of the statistical parameters including central tendency (e.g. means) or other basic estimates (e.g. regression coefficient) AND variation (e.g. standard deviation) or associated estimates of uncertainty (e.g. confidence intervals)
☐	☒	For null hypothesis testing, the test statistic (e.g. F, t, r) with confidence intervals, effect sizes, degrees of freedom and P value noted<br>*Give P values as exact values whenever suitable.*
☒	☐	For Bayesian analysis, information on the choice of priors and Markov chain Monte Carlo settings
☒	☐	For hierarchical and complex designs, identification of the appropriate level for tests and full reporting of outcomes
☒	☐	Estimates of effect sizes (e.g. Cohen's d, Pearson's r), indicating how they were calculated

Our web collection on statistics for biologists contains articles on many of the points above.

Software and code

Policy information about availability of computer code

Data collection	No software was used.
Data analysis	BWA (v.0.7.10-r789), SAMtools (v1.1) and REDitools (v1.0.4) was used for targeted base editing analysis. FastQC (v.0.11.9), fastp (0.20.1), bwa-mem2 (2.2.1) and GATK (4.3.0.0) were adopted for mitochondrial DNA off-target analysis. The coverage was evaluated by sambamba (v 0.6.6). Bowtie2 (2.4.5), Bedtools (v2.30.0) and GATK (4.3.0.0) were adopted for nuclear DNA off-target analysis.

For manuscripts utilizing custom algorithms or software that are central to the research but not yet described in published literature, software must be made available to editors and reviewers. We strongly encourage code deposition in a community repository (e.g. GitHub). See the Nature Portfolio guidelines for submitting code & software for further information.

Data

Policy information about availability of data

All manuscripts must include a data availability statement. This statement should provide the following information, where applicable:

- Accession codes, unique identifiers, or web links for publicly available datasets
- A description of any restrictions on data availability
- For clinical datasets or third party data, please ensure that the statement adheres to our policy

All data and materials presented in this manuscript are available from the corresponding author (W.W.) upon reasonable request. Raw data of off-target analysis are

available as a BioProject with Project ID PRJCA016204 in CNCB-NGDC database. The crystal structure of MutH interacting with unmethylated 5'-GATC-3' is available in PDB database (ID: 2AOQ). The confirmed human diseases related mtDNA mutations in Fig.6l are calculated from the MITOMAP database.

Human research participants

Policy information about studies involving human research participants and Sex and Gender in Research.

Reporting on sex and gender	Not applicable.
Population characteristics	Not applicable.
Recruitment	Not applicable.
Ethics oversight	Not applicable.

Note that full information on the approval of the study protocol must also be provided in the manuscript.

Field-specific reporting

Please select the one below that is the best fit for your research. If you are not sure, read the appropriate sections before making your selection.

☒ Life sciences ☐ Behavioural & social sciences ☐ Ecological, evolutionary & environmental sciences

For a reference copy of the document with all sections, see nature.com/documents/nr-reporting-summary-flat.pdf

Life sciences study design

All studies must disclose on these points even when the disclosure is negative.

Sample size	In this study, targeted base editing of mtDNA was done with three independent biological replicates performed in parallel, and the number of replicates was listed in the text and figure legends. Three or more of independent experiments performed in off-target analysis were described in Statistics and Reproducibility. Three independent replicates were performed with similar results in agarose electrophoresis. Three independent biological replicates met the needs of the unparied two-tailed Student's T test, so we performed three or more independent replicates of each experiment
Data exclusions	No data were excluded.
Replication	The number of replications is always mentioned in text, methods and figure legends. All attempts at replication were successful.
Randomization	All experiments are randomly allocated.
Blinding	Yes

Reporting for specific materials, systems and methods

We require information from authors about some types of materials, experimental systems and methods used in many studies. Here, indicate whether each material, system or method listed is relevant to your study. If you are not sure if a list item applies to your research, read the appropriate section before selecting a response.

Materials & experimental systems

n/a	Involved in the study	
☒	☐	Antibodies
☐	☒	Eukaryotic cell lines
☒	☐	Palaeontology and archaeology
☒	☐	Animals and other organisms
☒	☐	Clinical data
☒	☐	Dual use research of concern

Methods

n/a	Involved in the study	
☒	☐	ChIP-seq
☒	☐	Flow cytometry
☒	☐	MRI-based neuroimaging

Eukaryotic cell lines

Policy information about cell lines and Sex and Gender in Research

Cell line source(s)	HEK293T (ATCC, CRL-3216) were obtained from C. Zhang's laboratory (Peking University). RPE1 (ATCC , CRL-4000) purchased from Edigene. NCI-H1299 (ATCC, CRL-5803), Huh7 (JCRB, JCRB0403), and MCF7 (ATCC, HTB-22) purchased from Pricella. The GM10742 (LCL from B-Lymphocyte) purchased from Coriell Institute.
Authentication	STR analysis was used for cell line authentication.
Mycoplasma contamination	All cells tested negative for mycoplasma contamination.
Commonly misidentified lines (See ICLAC register)	No commonly misidentified cell lines were used.
